# Cell biological mechanisms of activity-dependent synapse to nucleus translocation of CRTC1 in neurons

**DOI:** 10.3389/fnmol.2015.00048

**Published:** 2015-09-04

**Authors:** Toh Hean Ch'ng, Martina DeSalvo, Peter Lin, Ajay Vashisht, James A. Wohlschlegel, Kelsey C. Martin

**Affiliations:** ^1^Lee Kong Chian School of Medicine, Nanyang Technological UniversitySingapore, Singapore; ^2^School of Biological Sciences, Nanyang Technological UniversitySingapore, Singapore; ^3^Department of Biological Chemistry, University of California, Los AngelesLos Angeles, CA, USA; ^4^Department of Neurology and Neuroscience, Stanford UniversityPalo Alto, CA, USA; ^5^Department of Psychiatry and Biobehavioral Sciences, University of California, Los AngelesLos Angeles, CA, USA

**Keywords:** CREB, CRTC1, learning and memory, synapse to nucleus signaling, synaptic plasticity, transcription-dependent plasticity, active transport

## Abstract

Previous studies have revealed a critical role for CREB-regulated transcriptional coactivator (CRTC1) in regulating neuronal gene expression during learning and memory. CRTC1 localizes to synapses but undergoes activity-dependent nuclear translocation to regulate the transcription of CREB target genes. Here we investigate the long-distance retrograde transport of CRTC1 in hippocampal neurons. We show that local elevations in calcium, triggered by activation of glutamate receptors and L-type voltage-gated calcium channels, initiate active, dynein-mediated retrograde transport of CRTC1 along microtubules. We identify a nuclear localization signal within CRTC1, and characterize three conserved serine residues whose dephosphorylation is required for nuclear import. Domain analysis reveals that the amino-terminal third of CRTC1 contains all of the signals required for regulated nucleocytoplasmic trafficking. We fuse this region to Dendra2 to generate a reporter construct and perform live-cell imaging coupled with local uncaging of glutamate and photoconversion to characterize the dynamics of stimulus-induced retrograde transport and nuclear accumulation.

## Introduction

Long-lasting forms of synaptic plasticity, including those underlying long-term memory, require new transcription for their persistence (Kandel, 2001; Alberini, 2009; Leslie and Nedivi, 2011). While neurons are specialized for rapid communication between compartments via electrochemical signaling, activity-dependent transcription is also regulated by the transport of soluble signaling molecules from stimulated synapses to the nucleus (Ch'ng et al., 2012; Karpova et al., 2013). Neurons are highly polarized cells that elaborate processes whose lengths can exceed that of the soma by orders of magnitude. The long-distance transport of signals from stimulated synapses to the nucleus thus requires active, regulated transport mechanisms to couple synaptic stimulation with transcription.

The regulated nuclear import of dendritically and/or synaptically localized transcriptional regulators serves as one means of directly coupling synaptic events with gene expression in the nucleus. Recent studies describe a role for the synapse to nucleus translocation of CREB-regulated transcriptional coactivator 1 (CRTC1) in regulating gene expression during long-term potentiation (LTP) of rodent hippocampal synapses (Zhou et al., 2006; Kovács et al., 2007; Ch'ng et al., 2012; Nonaka et al., 2014). CRTC1 was originally identified in an *in vitro* screen aimed at identifying proteins that enhance the transcriptional activity of CREB in non-neuronal cells (Iourgenko et al., 2003; Screaton et al., 2004). It has diverse functions in the brain including modulation of memory in rodents and flies (Zhou et al., 2006; Sekeres et al., 2012; Hirano et al., 2013; Nonaka et al., 2014), entrainment of circadian rhythms (Jagannath et al., 2013), neuroprotection during ischemia (Sasaki et al., 2011), and regulation of cocaine-induced plasticity (Hollander et al., 2010). Both Huntington's and Alzheimer's diseases have also been linked with CRTC1-mediated activation of CREB transcription of specific target genes (Jeong et al., 2012; Saura, 2012).

We previously reported that CRTC1 undergoes activity-dependent rapid translocation from distal dendrites to the nucleus during long-term plasticity of hippocampal neurons (Ch'ng et al., 2012). We showed that CRTC1 translocation required glutamate receptor activation, involved calcineurin-dependent dephosphorylation of CRTC1, and was critical to the activity-dependent expression of several CREB target genes (Ch'ng et al., 2012). These findings raised many questions about the mechanisms mediating the long-distance retrograde transport of CRTC1 from synapse to nucleus. The experiments described in this study are aimed at addressing these questions. Of note, while previous studies have examined the transport of vesicles and organelles in axons and dendrites (van den Berg and Hoogenraad, 2012; Maeder et al., 2014), much less is known about the cell biological mechanisms mediating the long-distance retrograde transport of soluble molecules in neurons. As such, our study provides insights into not only the transport of CRTC1, but also more broadly the retrograde transport of soluble molecules within dendrites.

We first examine the specific types of stimuli that trigger synapse to nuclear import of CRTC1 and find that it requires activation of glutamate receptors, calcium influx specifically though L-type but not P/Q or N-type calcium channels, and local rather than bulk elevations in intracellular calcium. We then show that CRTC1 is actively transported along microtubules by the dynein motor protein. Using protein domain analysis, we show that the N-terminal 270 amino acids of CRTC1 are sufficient for regulated nucleocytoplasmic localization, and within this region identify a non-canonical nuclear localization signal that is necessary and sufficient for CRTC1 nuclear import. We generate Ser to Ala mutations at three highly conserved Ser residues within the N-terminal third of CRTC1, and show that dephosphorylation of all three residues is necessary and sufficient for dissociation from 14-3-3ε at the synapse and for nuclear accumulation. Finally, we create a viral reporter construct consisting of the N-terminal third of CRTC1 fused to the photoconvertible fluorescent protein dendra2, and perform live cell imaging to visualize and characterize the dynamics of synapse-specific activation of CRTC1 nuclear import.

## Materials and methods

### Plasmids and antibodies

The CMV-mCherry-dynamitin expression vector was kindly shared by M. Meffert (Johns Hopkins, MD; Shrum et al., 2009) while the mCherry plasmid was a gift from R.Y. Tsien (UC San Diego, CA). The 4xGFP construct was a gift from W. Hampe (UMC Hamburg-Eppendorf, Hambug; Seibel et al., 2007). Commercial plasmids include Dendra2 (Evrogen) and CRTC1 (Open Biosystems, Huntsville, AL). Antibodies used in all these experiments include: rabbit polyclonal antibodies against CRTC1 (Bethyl, Montgomery, TX and Proteintech, Chicago, IL), pCRTC1(S151; Bethyl) Dendra2 (Evrogen, Moscow, Russia), TUJ1 (Covance, Princeton, NJ), Dynein heavy chain (Santa Cruz, Dallas, TX), and phosphoCREB-S133 (Cell Signaling); mouse monoclonal antibodies against PSD95 (Thermoscientific, Rockford, IL), synapsin1 (Millipore, Billerica, MA), CamKIIα (Millipore), HA-epitope (Sigma), GAPDH (Fitzgerald, Acton, MA), GFP (Clontech, Mt. View, CA), GAD67 (Millipore), and KPNB1 (ABR, Golden, CO); polyclonal chicken antibody against MAP2 (Phosphosolutions, Aurora, CO) and synaptotagmin (Chemicon, Temecula, CA). All secondary antibodies are conjugated to Alexa dyes (488, 546, 555, 568, and 633; Invitrogen).

### Viruses and expression constructs

Lentiviral packaging constructs bearing the L22 (*Camk2*α) promoter were kind gifts from Pavel Osten's lab (Dittgen et al., 2004). All production of lentiviral particles is as described in Dittgen et al. (2004). Lentiviral transduction of neurons was carried out in a reduced volume for 24 h before replacement with conditioned medium. The integrated constructs were allowed to express for at least 6 d prior to experiments.

### Dissociated neuron cultures protocols and pharmacological treatments

All experiments were performed using approaches approved by the UCLA Institutional Animal Care and Use Committee. Unless otherwise stated, all experiments in this report uses mature hippocampal neurons (DIV 21–28) dissected from newborn (P0) rats and plated on poly-DL-lysine (0.5 mg/ml) coated cover slips (Carolina Biologicals, Burlington, NC). Only the siRNA knockdown of dynein heavy chain experiment utilizes cultured mouse neurons. A defined serum-free media was used to culture the neurons: Neurobasal media, (Invitrogen, Carlsbad, CA); β-mercaptoethanol (Sigma, St. Louis, MO); monosodium glutamate (Sigma); B27 (Invitrogen); GlutaMAXI (Invitrogen). For most of the experiments, unless otherwise indicated, neurons were incubated with various pharmacological agents in conditioned neuronal media, in a 37°C, 5% CO_2_ incubator for the appropriate amount of time before cells were either fixed for immunocytochemistry or lysates were collected for immunoblots. For temperature-dependence experiment, neurons were maintained in parallel incubator kept at a constant 10°C. For neuronal transfection of plasmids, we employ a calcium chloride transfection protocol modified as previous described (Jiang and Chen, 2006). For receptor antagonist treatments (APV, NBQX, ifenprodil, etc.), unless otherwise stated, the cultured neurons were usually pre-treated with the antagonist for 30–60 min prior to stimulation. Unless otherwise stated, bicuculline (BIC) stimulation of neurons lasts for 15 min while AMPA or NMDA treatments lasts for 10 min followed by a washout and recovery for 5 min prior to processing for immunocytochemistry. The following pharmacological agents were used: bicuculline (BIC, 40 μM; Sigma), forskolin (FSK, 25 μM; Calbiochem, San Diego, CA), tetrodotoxin (TTX, 1 μM; Tocris, Ellisville, MO), APV (100 μM; Tocris), cyclosporin A (CsA, 5 μM; Sigma), nocodazole (NDZ, 0.1 μM or 20 μM; Tocris), BAPTA-AM (25 μM, Tocris), EGTA-AM (25 mM or 100 mM; Invitrogen), nimodipine (NIM, 10 μM; Tocris), ω-conotoxin (2 μM; Tocris), and ω-agatoxin (0.1 μM; Tocris), MNI-caged glutamate (0.2 mM; Tocris), NBQX (100 μM; Tocris); Trolox (10 nM; Tokyo Chemical Industry, Tokyo, Japan), ifenprodil (IFP, 50 μM; Tocris), MK801 (50 μM; Tocris), NMDA (20 μM; Tocris), AMPA (25 μM; Tocris), SN50 and SN50M (Enzo Lifesciences).

### Immunocytochemistry

All cells were fixed at room temperature with parafomalydehyde (3.2%) for 10 min, permeabilized with 0.1% Triton-X 100 (Calbiochem) for 5 min and blocked in 10% goat-serum for 30 min. Neurons were then incubated in primary antibodies either for 4 h at room temperature or overnight at 4°C. Secondary antibodies and Hoechst nuclear dye (Invitrogen) were incubated at room temperature at 1:2000 (2 μg/ml) dilution for 2 h. All antibodies were diluted in 10% goat serum and coverslips were mounted with aqua/polymount (Polysciences, Warrington, PA).

### Calcium chelators (BAPTA-AM and EGTA-AM) and calcium channel antagonists

Hippocampal neurons were pre-treated with TTX (1 μM) in the presence of DMSO, BAPTA-AM (25 mM), EGTA-AM (25 mM or 100 mM) in conditioned media. After 30 min of pretreatment, neurons were was washed with conditioned media and allowed to recover for 10 min before being stimulated in Tyrode's solution with varying concentrations of KCl (5 mM [5K^+^] or 40 mM [40K^+^]) for 5–7 min. Neurons were then washed and allow to recover in regular Tyrode's solution (5 mM) for another 5 min before being fixed and stained for immunocytochemistry. TTX (1 μM) was present in all media and solutions throughout pre-treatment, stimulation and recovery periods. Similarly, for calcium channel antagonists nimodipine (10 μM; NIM), ω-conotoxin (2 μM; CTX), and ω-agatoxin (0.1 μM; AGA), neurons were pre-treated for 30 min before being stimulated in Tyrode's solution containing elevated levels of KCl (40 mM) briefly for 5–7 min, washed and recovered in regular Tyrode's solution (5 mM) for another 5 min prior to fixation. TTX was present throughout the entire treatment protocol. Tyrode's solution (140 mM NaCl; 10 mM HEPES pH 7.3; 5 mM KCl; 3 mM CaCl_2_; 1 mM MgCl_2_; 10 mM glucose pH 7.35).

### Photomanipulation and analysis of CRTC1^270^ in neurons

Hippocampal neurons were transduced with virus expressing either Dendra2 or CRTC1^270^. After 1 week of expression, neurons were transferred on to a glass bottom live imaging setup containing a low Mg^2+^ Tyrode's solution (140 mM NaCl; 10 mM HEPES pH 7.3; 5 mM KCl; 3 mM CaCl_2_; 0.1 mM MgCl_2_; 10 mM glucose; 10 nM Trolox pH 7.35) with the appropriate reagents added as indicated for each experiment (TTX,1 μM; MNI-caged glutamate, 200 μM; DMSO). Neurons were allowed to recover on a heated stage assembly on a Zeiss LSM 700 scanning confocal microscope for approx. Ten to fifteen minutes prior to the first imaging session. Neurons with comparable levels of expression of fluorescent proteins were selected for imaging. We then selected two regions of interest (10 microns in length) in dendritic branches located roughly 100 μm away from the soma and exposed briefly to UV (405 nm) laser (dwell time: 51.2 ms) every 10th frame to photoconvert and photouncage glutamate. For each region of interest a total of 80 frames were captured per neuron. However, since the dimensions of each image capture varies slightly between neurons, the time it takes to capture 80 frames for each neuron will also have minor differences, with the average total time of image capture per neuron averaging between 13 and 15 min. To maintain consistency across the different time-lapse plots, we plotted the time elapsed in frames. Pharmacological reagents used during the experiments include APV (100 μM); NBQX (100 μM); EGTA-AM (25 μM); BAPTA-AM (25 μM); Nocodazole (0.1 μM 6 h pre-treatment prior to imaging). To analyze the data, the amount of Dendra2^red^ signal was quantified in the nucleus and the change in signal intensity over baseline was plotted over the total amount of frames acquired.

### Zeiss scanning confocal microscope LSM 700

Objectives: Plan Apochromat 63X 1.40 Oil DIC.

Temperature: 25 or 35°C (Zeiss Temp-control 37-2).

Media: Tyrode's solution for live imaging.

Lasers: 405, 488, 555, and 639 nm solid state lasers.

Data acquisition: Zen 2009.

### Local photoconversion and analysis of CRTC1^270^ in distal dendrites

Neurons were transduced and prepared for imaging as described in the previous section “photomanipulation of CRTC1^270^ in neurons.” However, we employed a Marianas spinning disc confocal system that is coupled to a Photometrics Evolve camera (Intelligent Imaging Innovations, Denver, CO) for high-speed image acquisition since this was required to capture the translocation of Dendra2-CRTC1 within the dendrite. Images were acquired within a humidified environmental chamber for regulated temperature control. A 2.25 cm^2^ region of activation (ROA) was selected on a dendritic branch located approximately 50–70 μm away from the soma. Using a Vector scan module attached to the system, the selected ROA was briefly exposed to UV laser (50% intensity; 2 ms dwell; Supplemental Figure S4). A total of 100 frames (alternating 473 and 523 nm beam excitation) were acquired per imaging session (approximately 660 ms/frame). Pharmacological reagents used in the experiment include BIC (40 μM) and forskolin (25 μM). To calculate the average intensity of dendrites flanking the ROA, a series of regions were drawn bidirectionally to highlight 40 μm of dendrites both proximal and distal to the ROA. The average intensity of these 40 μm segments were quantified over time for all neuronal samples within the same experimental group. The group data is presented as a scatter plot of the average intensity of the entire 40 μm segment either proximal or distal to the ROA over time (as indicated by the x-axis showing frames captured over time). To plot the bias index, the average intensity of the distal segment is subtracted from the proximal segment and the difference of the value is plotted on a horizontal scatter plot for all time points collected in each experimental condition. A positive bias index indicated that the proximal segment has a higher average intensity compared to the distal segment while an average index value of zero indicates the overall average intensity over time is the similar in both branches of dendrites. The cumulative bias index calculates the average bias index for all the neurons in the same experimental group and plots the cumulative total over time.

### 3i Marianas spinning disc confocal system (CSU22 yokugawa scanner)

Objectives: Plan Apochromat 63X 1.40 Oil DIC.

Temperature: 35°C (Okolab temperature control chamber).

Media: Tyrode's solution for live imaging (methods and materials).

Lasers: 405, 488, and 561 nm solid state lasers.

Camera: Photometric Evolve EMCCD camera.

Data acquisition: Slidebook 5.0.

### Cell cultures, transfections and, siRNA treatment

All cell lines (HEK293T) were grown on Dulbecco's Modified Eagle Media (DMEM; Invitrogen) supplemented with 10% fetal bovine serum (HyClone, Logan, UT), and 1% Penicillin/Streptomycin (Invitrogen). Transfection of plasmids in cell lines was carried out with Lipofectamine 2000 (Invitrogen) using protocols recommended by the manufacturer. For siRNA treatment of human CRTC1 or GAPDH in HEK293T cells, we purchased custom designed siGENOME SMARTpool siRNA from Dharmacon (Lafeyette, CO). The SMARTpool siRNA (50 nM) was delivered into cells using Lipofectamine RNAiMAX (Invitrogen) reagent over the course of 72 h with refeeding of siRNA every 24 h and the replating and reseeding of cell after 48 h. For siRNA treatment of human importin β1 (50 nM; KPNB1; SMARTpool siRNA; Dharmacon) and GAPDH, HEK293T cells were incubated with KPNB1 siRNA for 48 h before expression plasmids bearing different 4xGFP fusion constructs were transfected into cells for another 12 h prior to immunocytochemistry or immunoblotting. For siRNA treatment of mouse neurons with Dynch1h, we purchased custom designed siGENOME SMARTpool Accell siRNA from Dharmacon. The Accell siRNA (1 μM) was delivered into neurons directly and allowed to incubate for 48–96 h. After the incubation period, cells were either fixed for immunocytochemistry or harvested in lysis buffer for analysis via Western blots.

### Image analysis and synapse quantification

We performed all nuclear to cytoplasmic ratio quantification using Slidebook v4.2-v5.5 (Intelligent Imaging Innovations). Briefly, raw confocal images taken using a 63X 1.4NA oil objective were imported into Slidebook. For each neuron, masks were manually drawn to highlight both the nucleus (based on Hoechst staining) and the entire cell body (based on MAP2 staining). A simple subtraction routine was performed for these two masks to obtain a separate mask for the cytoplasm. The nuclear to cytoplasmic ratio of individual neurons were calculated based on the average intensity of staining as defined by the nuclear and cytoplasmic masks for each image. All data points were then plotted using Prism Graphpad. Unless otherwise stated, all data sets are presented as mean ± SEM either as bar graphs or scatter plots and, all *p*-values were determined using One-Way analysis of variance (ANOVA) with Bonferroni's Correction *post-hoc* test. To analyze synaptic integrity, hippocampal neurons were transduced with an AAV expressing GFP being driven under the neuron-specific synapsin promoter. After 1 week of expression, neurons were incubated in NDZ (0.1 μM) for 6 h before being stimulated and processed for immunocytochemistry using antibodies against PSD95, Synapsin and MAP2. To quantify for the number of synapses, Slidebook was used to isolate only PSD95-positive and synapsin-positive puncta based on signal intensity, size exclusion, and overlapping fluorescence intensities. The stringent selection criteria to increase signal to noise for each puncta likely resulted in undersampling. To identify synapses, only PSD95 and synapsin masks that overlapped were considered as a positive score. Student's *t*-test (two-tailed, unpaired) was used as a statistical analysis.

### Gene expression in CRTC1^270^ transduced hippocampal neuronal cultures

Hippocampal cultures were transduced with lentivirus expressing CRTC1^270^ and 1 week post-transduction, neurons were pre-treated with TTX (1 μM) for another 3 h before either being continuously maintained in TTX or the TTX was withdrawn, washed and replaced with regular conditioned media. The neurons were allowed to recover for 30 min before RNA was extracted with TRIzol (Invitrogen), column purified with RNeasy kit (Qiagen, Valencia, CA), and the concentration determined via the Nanodrop (Thermo Scientific). For RT-PCR, we generated cDNA using reverse transcriptase and poly dT primers (Invtirogen). We then conducted quantitative real time PCR experiments (Stratagene Mx3000P), with SYBR Green Master Mix (Invitrogen) and specific primer pairs (Operon, Huntsville, AL) listed below. ΔΔCt values were calculated for all raw qPCR results by normalizing against mock, untreated controls and corrections for loading error we made against ΔΔCt values for HPRT1, a non-activity-dependent gene. Primer pairs used include crtc1 (5-tggacagagtatatcgtgagcg; 5-catgcttgtctactgacaggg), arc (5-ccgtcccctcctctcttga; 5-aaggcacctc ctctttgtaatcctat), hprt1 (5-agtcccagcgtcgtgattag; 5-ccatctccttcatgacatctcg), btg2 (5-tcc tgaggactcggggctgc; 5-gcgatagccggagcccttgg), cfos (5-tcccagctgcactacctatacgt; 5-tg cgcagctagggaagga), cyr61 (5-aactcggagtgccgcctggt; 5-gccgcagtatttgggccgg), zif268 (5-attgatgtctccgctgcagat; 5-gtagttgtccatggtgggtga), and Dendra2 (5-ggaattaacctgatcaagga; 5-tggaagaagcagtcgccctc).

### GST-14-3-3ε pulldowns of CRTC1^270^ fusion proteins and western blots

Cortical neuron cultures (DIV 21–28) were transduced with lentiviruses expressing either full length or serine to alanine CRTC1^270^ mutants. After 1 week. post-transduction, neuronal lysates were collected (25 mM Tris, pH7.4; 137 mM NaCl; 1% NP40; 10% glycerol; protease and phosphatase inhibitors; Ballif et al., 2006) and subjected to pulldowns using purified GST-14-3-3 ε bound on glutathione beads. The beads were then washed extensively, resuspended in sample buffer and analyzed via immunoblots using either conventional or PhosTAG acrylamide gels. For Western blotting, all samples were loaded on 8–10% bisacrylamide minigels (Biorad) and ran on standard PAGE buffers. After appropriate amount of time, proteins bands were transferred onto PVDF (Amersham) before being incubated with antibodies for detection. All western blot images were obtained with Odyssey Imaging System (LI-COR Biosciences) and quantified using Image Studio.

### Mass spectrometry

Four confluent 10 cm dishes of Neuro-2A cells were transfected with HA-CRTC1 plasmid using Lipofectamine-2000. After 16 h, cells were washed twice in ice cold PBS and lysed cells in lysis buffer (150 mM NaCl, 50 mM Tris (pH 8.0), 1% NP40) supplemented with protease and phosphatase inhibitors (Roche). The lysates were incubated on ice for 20 min with benzonase DNase (Millipore) to reduce viscosity, then centrifuged to clarify insoluble proteins. HA-CRTC1 was immunoprecipitated using pre-equilibrated EZview Red Anti-HA Affinity Gel (Sigma) for 2 h at 4°C with constant rotation. Beads were washed 3X in ice-cold lysis buffer, and eluted using 100 μl 8M urea. Samples were mixed with loading buffer, boiled and loaded onto NuPAGE Novex 4–12% Bis-Tris gradient gel. To purify HA-CRTC1 for mass spec, the gel was stained with Simply Blue SafeStain (Invitrogen) and CRTC1 was excised from gel slices, digested with either trypsin or chymotrypsin before fractionated online using a C18 reversed phase column, and analyzed by MS/MS on a Thermofisher LTQ- Orbitrap XL as previously described (Kaiser and Wohlschlegel, 2005; Wohlschlegel, 2009). MS/MS spectra were subsequently analyzed using the ProLuCID and DTASelect algorithms (Eng et al., 1994; Tabb et al., 2002). Phosphopeptides were identified using a differential modification search that considered a mass shift of +79.9663 on serines, threonines, and tyrosines. All phosphorylated sites reported in **Figure 7** exhibit an AScore of at least 90% confidence levels.

## Results

### Glutamate receptor and L-type calcium channel activation promote nuclear translocation of CRTC1

**Figure 1:**
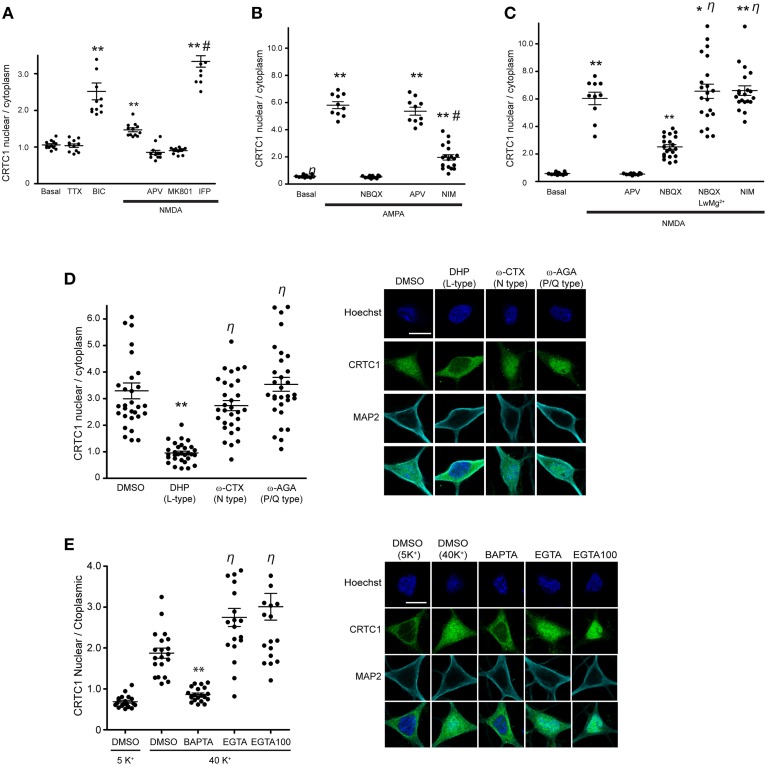
**CRTC1 nuclear translocation requires local calcium influx via glutamate receptors and L-type VGCCs**. **(A)** Nuclear to cytoplasmic ratio of CRTC1 in cultured neurons after stimulation with NMDA in the presence of different NMDA receptor antagonists, APV, MK801 or Ifenprodil (IFP) (^**^p < 0.001 relative to basal; ^#^*p* < 0.001 relative to NMDA). **(B,C)** Nuclear to cytoplasmic ratio of CRTC1 in cultured neurons pre-incubated with TTX and IFP, in conjunction with either NBQX, APV or nimodipine (NIM) before being stimulated with either AMPA or NMDA. Experiments were performed in either Tyrode's with regular MgCl_2_ (1 mM) or low MgCl_2_ (0.1 mM) (^**^p < 0.001 relative to basal; ^*^p < 0.001 relative to NBQX; ^#^*p* < 0.001 relative to AMPA; η: not significant relative to NMDA). **(D)** Confocal micrographs and nuclear to cytoplasmic ratio quantification of CRTC1 in neurons depolarized with KCl in the presence of NIM, conotoxin (ω-CTX), agatoxin (ω-AGA), or mock treated with DMSO (^**^p < 0.001 relative to DMSO; η: not significant relative to DMSO). **(E)** Membrane permeable calcium chelators BAPTA and EGTA (25 μM/100 μM) were incubated in neurons prior to depolarization with KCl (5K^+^ 5 mM; 40K^+^ 40 mM). Nuclear to cytoplasmic ratio of CRTC1 was quantified (^**^p < 0.001 relative to DMSO 40K^+^; η: not significant relative to DMSO 40K^+^). All scale bars = 10 μm.

We previously reported that activation of NMDA receptors was required for CRTC1 synapse to nucleus transport (Ch'ng et al., 2012) but did not characterize the type of NMDA receptor involved. Here, we asked whether GluN2B-containing NMDA receptors, which are expressed at highest levels neonatally, are involved by bath applying NMDA to cultured neurons to activate NMDA receptors while selectively blocking GluN2B-containing NMDA receptors with the inhibitor ifenprodil. In control experiments, we blocked with APV, which blocks all NMDA receptors or with MK801, an open channel, usage-dependent antagonist that only blocks activated NMDA receptors (Thompson et al., 2004). Immunocytochemical analysis revealed that MK801 and APV blocked NMDA-induced nuclear accumulation of CRTC1 and phosphorylation of CREB at serine 133, but that ifenprodil did not. In fact, stimulation with NMDA in the presence of ifenprodil triggered significantly more nuclear accumulation of CRTC1 and phosphorylation of CREB at serine 133 than did NMDA alone, suggesting that activation of GluN2B-containing NMDA receptors functions to repress CRTC1 synapse to nucleus import (Figure 1A and Supplementary Figures S1A,B).

Next, we focused on the AMPA receptor, which plays a crucial function in various forms of learning-related neuronal plasticity (Kessels and Malinow, 2009). Inhibition of NMDA receptors with APV greatly reduced but did not completely abolish bicuculline (BIC) -induced CRTC1 translocation (Ch'ng et al., 2012), indicating that other synaptic glutamatergic receptors contribute to CRTC1 nuclear localization. Selective activation of AMPA receptors with AMPA triggered robust CRTC1 nuclear accumulation, which was significantly reduced by the AMPA receptor blocker NBQX (Figure 1B). The L-type voltage-gated calcium channel (VGCC) antagonist nimodipine also reduced AMPA receptor-mediated CRTC1 nuclear accumulation, suggesting that activation of synaptic AMPA receptors produces sufficient local depolarization to activate VGCCs (Macías et al., 2001; Higley and Sabatini, 2012) and that the resulting influx of calcium contributes to CRTC1 nuclear import. AMPA-induced CRTC1 nuclear accumulation was not inhibited by APV, indicating that calcium influx through AMPA and L-type VGCC is sufficient to drive CRTC1 nuclear translocation.

We next tested the hypothesis that AMPA receptor activation might enhance CRTC1 nuclear entry by activating synaptic NMDA receptors. Toward this end, we preincubated neurons with NBQX (to block AMPA receptors), TTX (to block action potentials) and ifenprodil (to block GluN2B-containing NMDA receptors) before briefly exposing neurons to low concentrations of NMDA. Under these conditions, NMDA-induced CRTC1 nuclear accumulation was significantly diminished, consistent with a role for AMPA receptors in NMDA receptor-mediated regulation of CRTC1. To explain these results, we hypothesized that bath application of NMDA triggered sufficient depolarization of neurons to enhance glutamate release, leading to AMPA receptor activation, which further depolarizes the neurons to relieve the Mg^2+^ block in NMDA receptors, thereby increasing the effect of NMDA. To test the idea that depolarization induced by AMPA receptor activation contributes to the NMDA-induced translocation by helping to relieve the voltage-dependent Mg^2+^ block of the NMDA receptor channel, we reduced the concentration of Mg^2+^ ions in the Tyrode's solution by 10-fold during NMDA stimulation and showed that CRTC1 translocation no longer required AMPA receptor function, as it was not blocked by NBQX (Figure 1C). These results indicate that if the post-synaptic compartment is sufficiently depolarized to relieve the Mg^2+^ block in the NMDA receptor, then calcium influx through the NMDA receptor is adequate to drive CRTC1 nuclear translocation.

In addition to inhibiting CRTC1 nuclear import induced by incubation with AMPA, we previously showed that the L-type VGCC antagonist nimodipine also blocked CRTC1 nuclear import induced by incubation with the GABA_A_ receptor antagonist bicuculline (Ch'ng et al., 2012). To test whether the N-type or P/Q type VGCCs were also required for CRTC1 synapse to nucleus import, we depolarized neurons with KCl in the presence of conotoxin to block N-type VGCCs, agatoxin to block P/Q-type VGCCs, or nimodipine to block L-type VGCCs. As shown in Figure 1D, only inhibition of L-type VGCCs with nimodipine blocked KCl-induced CRTC1 translocation. Consistent with previously published reports, we also found that only L-type VGCCs were required to trigger depolarization-induced increase in pCREB S133 immunoreactivity (Supplementary Figure S1C; Wheeler et al., 2012).

To further characterize the source of calcium entry required for stimulus-induced CRTC1 nuclear import, we assayed the effect of both high affinity, fast-acting (BAPTA) and low affinity and slower-acting (EGTA) calcium chelators. BAPTA rapidly suppresses local elevations in calcium near their source of entry at the plasma membrane while EGTA has a 100-fold slower on rate and thus blocks bulk cytosolic elevations in calcium (Neher and Almers, 1986; Deisseroth et al., 1996). We found that BAPTA, but not EGTA completely blocked the nuclear accumulation of CRTC1 and phosphorylation of CREB at Serine 133 induced by KCl depolarization (Figure 1E and Supplementary Figure S1D). Together, these findings indicate that the local influx of calcium produced by calcium influx through synaptic NMDA and AMPA glutamate receptors and L-type VGCCs contribute to CRTC1 nuclear translocation.

### CRTC1 synapse to nuclear transport involves active, dynein-dependent movement along microtubules

**Figure 2:**
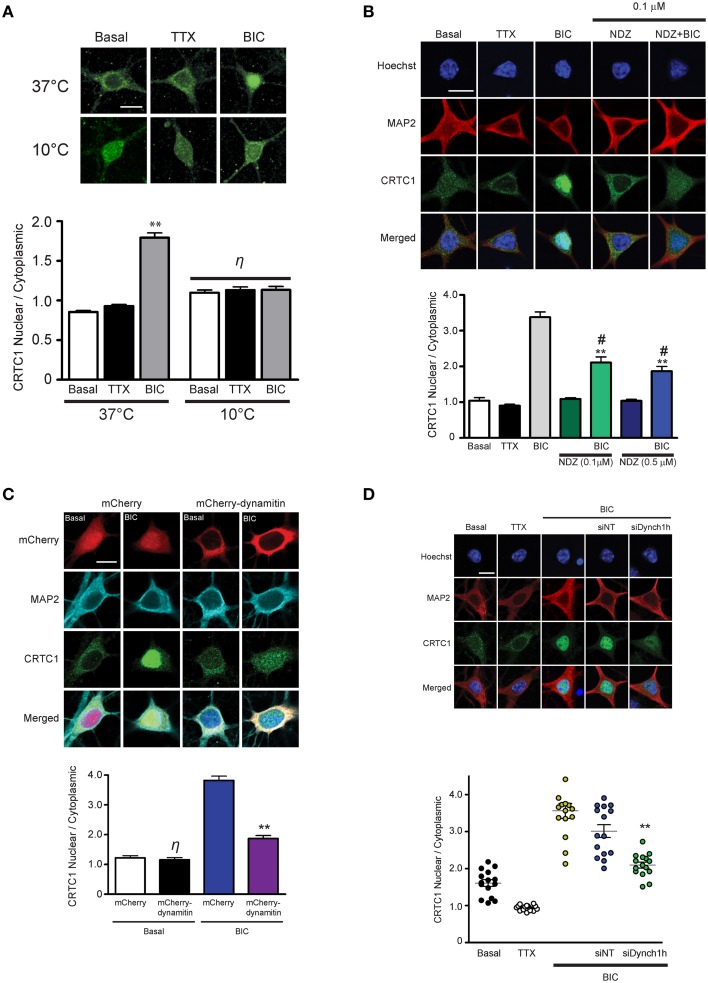
**Nuclear accumulation of CRTC1 involves active, dynein-mediated transport along microtubules**. **(A)** Neurons were stimulated with either TTX or BIC in incubators at 37°C or 10°C. CRTC1 nuclear to cytoplasmic ratio was quantified (^**^p < 0.001 relative to basal; η: not significant). **(B)** Neurons were incubated with nocodazole (NDZ, 0.1 μM or 0.5 μM) to depolymerize microtubules prior to BIC stimulation (^**^p < 0.001 relative to non-BIC samples ^#^*p* < 0.001 relative to BIC). **(C)** Neurons expressing either mCherry or mCherry-dynamitin were stimulated with BIC. Endogenous expression of CRTC1 in the nucleus and cytoplasm of mCherry expressing neurons were quantified (^**^p < 0.001 relative to mCherry only samples; η: not significant relative to basal mCherry). **(D)** Mouse hippocampal neurons (21–28 DIV) were incubated with dynein heavy chain siRNA (siDynch1h) or non-targeted control (siNT) followed by stimulation with TTX or BIC (^**^p < 0.001 relative to all other BIC samples). All scale bars = 10 μm.

In our previous experiments, the rapid nuclear accumulation of CRTC1 following synaptic stimulation suggested that soluble CRTC1 must be actively transported to the nucleus. To determine how CRTC1 is transported from distal stimulated synapses to the nucleus, we asked whether it required active transport, which, unlike passive diffusion, can be blocked at 10°C (Talcott and Moore, 1999; Wiegert et al., 2007). Stimulation of neurons with BIC at 10°C or 37°C revealed that CRTC1 nuclear accumulation was blocked at lower temperatures (Figure 2A), consistent with an active transport process. We then asked whether microtubules are required for the transport by depolymerizing microtubules with low concentrations of nocodazole and found that this significantly inhibited the nuclear accumulation of CRTC1 in response to BIC (Figure 2B) without compromising the viability, morphology or synapse density of the neurons (Supplementary Figures S2A,B).

We next asked whether CRTC1 nuclear import was mediated by the microtubule-based retrograde motor protein dynein. In initial experiments, we specifically disrupted cytoplasmic dynein function by overexpressing dynamitin, which functions as a dominant negative by triggering the disassembly of dynactin, a multiprotein complex required for dynein-based movement (Melkonian et al., 2007; Shrum et al., 2009). Dynamitin overexpression in neurons significantly decreased nuclear CRTC1 accumulation following BIC stimulation (Figure 2C). To complement these experiments, we used siRNA to knockdown the expression of dynein heavy chain (Supplementary Figure S2C), and found that this also significantly decreased BIC-induced CRTC1 nuclear accumulation as compared to BIC-stimulated control neurons incubated with non-targeted siRNA (Figure 2D). Together, these data indicate that dynein mediates the long-distance retrograde transport of CRTC1 along microtubules.

### CRTC1 encodes an arginine-rich nuclear localization signal

**Figure 3:**
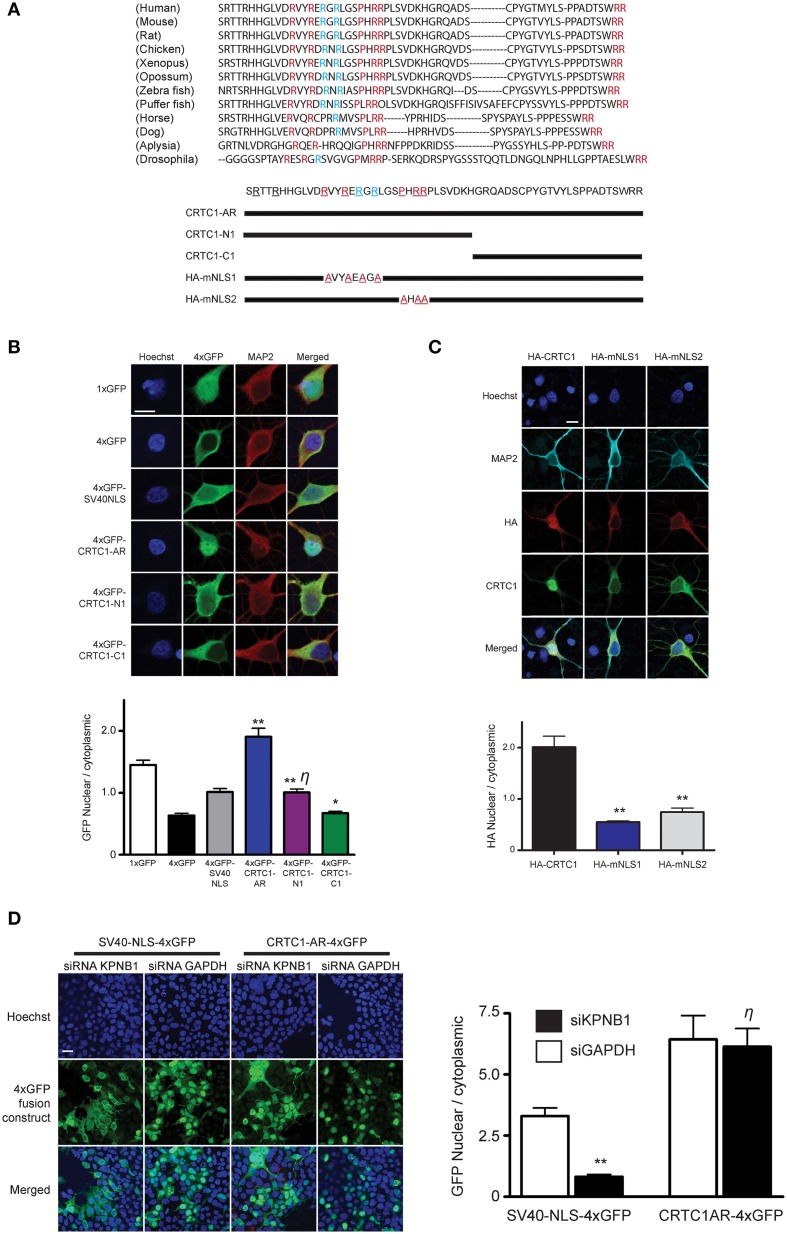
**Characterization of CRTC1's arginine-rich NLS. (A)** A 57 amino acid fragment (S92-R148) in CRTC1 with sequence identity (red) and similarity (blue) across species. Expression constructs described below. **(B)** As diagramed in **(A)**, the full length arginine-rich fragment (CRTC1-AR) as well as CRTC1-N1, CRTC1-C1 and SV40NLS control fragment fused to 4xGFP were expressed in neurons. GFP signal in the nucleus and cytoplasm were quantified (^**^p < 0.001 relative to 4xGFP; ^*^ not significant relative to 4xGFP; η: not significant relative to SV40NLS). **(C)** Neurons expressing full length HA-tagged CRTC1 and alanine mutations in two arginine-rich clusters (HA-mNLS1 and HA-mNLS2, as diagramed in **A**) were stimulated with BIC and immunostained with antibodies against HA epitope (^**^p < 0.001 relative to HA-CRTC1). **(D)** HEK293T cells were treated with siRNA against KPNB1 (Importin β1) or GAPDH before being transiently transfected with SV40NLS-4xGFP or CRTC1-AR-4xGFP. The nuclear to cytoplasmic ratio of GFP was quantified (^**^p < 0.001 relative to siGAPDH; η: not significant relative to siGAPDH). Scale bars = 10 μm.

Having determined the source of calcium entry at the synapse that triggers CRTC1 synapse to nuclear import, and shown that CRTC1 retrograde transport is active and occurs along microtubules in a dynein-dependent manner, we next focused on the signals which regulate CRTC1 transport into the nucleus. Nuclear import of proteins larger than 40–60 kD is typically facilitated by transport proteins that recognize nuclear localization signals (NLSs), which are often comprised of short stretches of basic amino acids (Lange et al., 2007). Analysis of the CRTC1 primary amino acid sequence revealed three highly conserved clusters of basic residues near the amino-terminus of the protein (Figure 3A). To determine whether these amino acid clusters comprised an NLS, we fused a 57 amino-acid sequence (aa 92–148) from CRTC1 that contained all three arginine-rich clusters (CRTC1-AR) to four tandem copies of GFP (4xGFP, Figure 3B). While 4xGFP localized exclusively to the cytoplasm of neurons, fusion to CRTC1-AR promoted robust nuclear accumulation of 4xGFP (Figure 3B). The amount of nuclear accumulation was greater than the nuclear accumulation observed when 4xGFP was fused to the canonical SV40 NLS. To identify the minimal region required for nuclear translocation, we further divided CRTC1-AR into two sub-fragments, CRTC1-N1 (amino acids 103–135), which contained two clusters of arginine residues, and CRTC1-C1 (amino acids 135–147). As shown in Figure 3B, only CRTC1-N1 fragment was able to confer accumulation in the nucleus of neurons, although to a lesser extent than full length CRTC1-AR, while the CRTC1-C1 fragment remained cytoplasmically localized. We also tested these sequences in HEK293T cells, and found that the full length CRTC1-AR and the CRTC1-N1 fragment both promoted nuclear import of CRTC1 (Supplementary Figure S3A).

To probe the necessity of the NLS in mediating the nuclear import of CRTC1, we generated two HA epitope-tagged full-length CRTC1 alanine mutants. In the first (HA-mNLS1), we mutated four highly conserved arginines to alanines (R103A, R106A, R108A, and R110A). In the second mutant (HA-mNLS2), we mutated one proline and two arginine residues to alanine (P114A, R116A, and R117A). Neither of these mutants disrupted phosphorylation of CRTC1 at serine residue 151, a site that has been shown to regulate nucleocytoplasmic trafficking in non-neuronal cells (Supplementary Figure S3B; Screaton et al., 2004). However, neither of these mutants were efficiently imported into the nucleus upon BIC stimulation, indicating that these particular conserved residues constitute essential components of the NLS (Figure 3C).

To determine if the classical nuclear import pathway was involved in CRTC1 nuclear entry (Thompson et al., 2004; Jeffrey et al., 2009), we used siRNA to knock down importin β1 (KPNB1) expression to 45% of baseline values (using GAPDH siRNA knockdown as controls) in HEK293T cells (Figure 3D and Supplementary Figure S3C). While the knockdown blocked the nuclear import of a canonical NLS (4xGFP-SV40), it did not inhibit the nuclear translocation of 4xGFP-tagged CRTC1-AR (Figure 3D). We also inhibited the classical nuclear import pathway using cell-permeable inhibitor peptides that mimic the NLS of NFκB SN50 (Lai et al., 2008) and did not detect any effect on BIC-induced CRTC1 nuclear translocation (Supplementary Figure S3D). Finally, we overexpressed 4xGFP-tagged CRTC1-AR and SV40 NLS in neurons (Supplementary Figure S3E) and showed that at high expression levels, only the CRTC1 arginine-rich NLS but not the SV40 NLS was able to inhibit nuclear translocation of endogenous CRTC1. Taken together, our experiments indicate that CRTC1 undergoes active transport into the nucleus in a manner that is independent of the classical importin α/β1-mediated pathway.

### The first 270 amino acids of CRTC1 are sufficient for stimulus-induced nucleocytoplasmic shuttling

**Figure 4:**
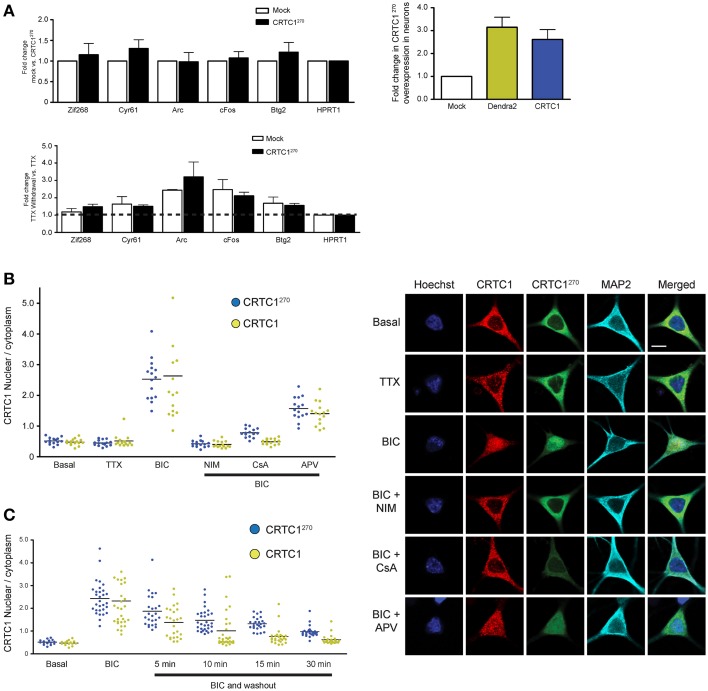
**The nuclear translocation of CRTC1^270^ is identical to endogenous full length CRTC1 and does not inhibit transcription of CREB target genes. (A)** Rat neurons expressing CRTC1^270^ or mock transduced were incubated with TTX. In half the samples, TTX was withdrawn to stimulate neuronal activity while the other half of the sample remained incubated in TTX. Total RNA was extracted after treatment and CREB target genes were analyzed via qPCR (*n* = 5 basal TTX; *n* = 3 stimulation by TTX withdrawal). The expression of CRTC1^270^ in neurons was also measured via qPCR using primers against Dendra2 or mouse CRTC1. **(B,C)** Neurons transduced with CRTC1^270^ were pretreated with APV, NIM, TTX, and cyclosporinA (CsA), stimulated with BIC, and fixed immediately or at the indicated time points after BIC washout. The amount of endogenous CRTC1 and CRTC1^270^ in the nucleus and cytoplasm were quantified. All paired quantifications of nuclear to cytoplasmic ratio between endogenous CRTC1 and CRTC1^270^ were not significant. Scale bars = 10 μm.

In the course of analyzing the different domains to identify the NLS in CRTC1, we observed that a fragment containing amino acids 1–270 of CRTC1 localized strongly to the nucleus of CHO cells. In contrast, the carboxy terminal fragment of CRTC1, from amino acids 271–630, localized exclusively to the cytoplasm (Supplementary Figure S4A). Further analysis in neurons show that fragment 1–270 localizes to dendrites and synapses in silenced neurons but underwent stimulus-induced translocation in a manner that was indistinguishable from full-length CRTC1 (Figure 4B and Supplementary Figure S4A). These findings indicated that the N-terminal 270 amino acids contained all of the signals required for regulated synaptic and nuclear localization.

We took advantage of this finding to develop a fluorescent reporter to monitor synapse to nucleus CRTC1 signaling by fusing the amino terminal fragment (aa 1–270) of CRTC1 to Dendra2, a photoconvertible fluorescent protein that switches from green (507 nm) to red (573 nm) emission following brief UV illumination. Unlike the full-length CRTC1, this fusion protein was small enough to package efficiently in a lentivirus using a *Camk2*α promoter to achieve modest levels of expression exclusively in excitatory neurons. Using this reporter, henceforth identified as CRTC1^270^, we were able to photoconvert a subpopulation of CRTC1 in dendrites of cultured hippocampal neurons (21–28 DIV) and track its stimulus-induced movement and nuclear accumulation (Chudakov et al., 2010). Long-term expression (>1 week) of the fusion protein in cultured neurons did not have any visible effect on neuronal viability.

To ensure that overexpression of CRTC1^270^ did not function as a dominant negative and thereby interfere with CREB-mediated activity-dependent transcription, we transfected neurons with CRTC1^270^ and used qPCR to test the induction of CREB target genes following TTX withdrawal (Saha et al., 2011). As shown in Figure 4A, overexpression of CRTC1^270^ did not alter the basal or the activity-induced expression of any CREB target genes, indicating that it did not have any dominant-negative activity.

We next set out to determine whether the dynamics of stimulus-induced synapse to nuclear import of CRTC1^270^ showed any differences from that of endogenous, full-length CRTC1. Toward this end, we cultured neurons and transduced them with lentivirus to express CRTC1^270^. We then stimulated with BIC, and performed immunocytochemistry with anti-dendra2 antibodies to visualize the localization of CRTC1^270^. In parallel, we performed immunocytochemistry with anti-CRTC1 antibodies to visualize CRTC1 localization in neurons that were not transduced with CRTC1^270^. As shown in Figure 4B, similar to endogenous full length CRTC1, BIC stimulated robust CRTC1^270^ nuclear translocation that required calcineurin, NMDA and L-type VGCC activation. Moreover, the kinetics of nuclear export of CRTC1^270^ following BIC stimulation was indistinguishable from that of full-length, endogenous CRTC1 (Figure 4C).

### Local uncaging of glutamate at synapses drives distally-localized CRTC1 into the nucleus

**Figure 5:**
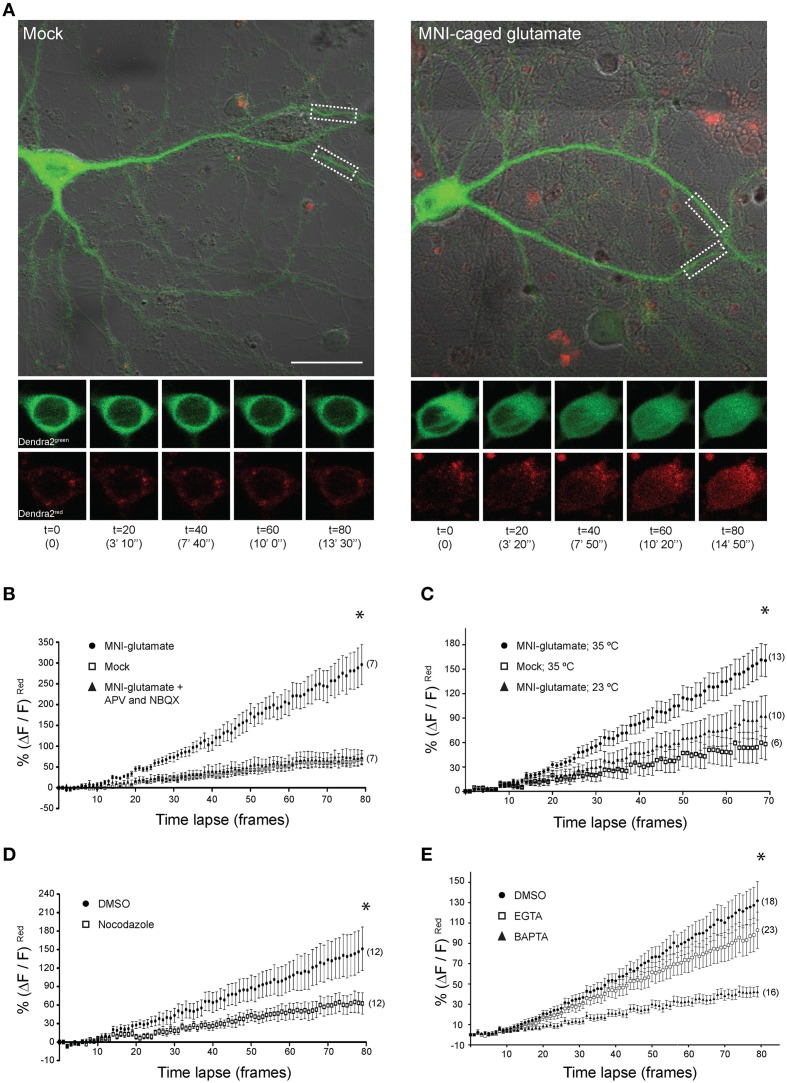
**Local uncaging of glutamate at synapses can drive distally-localized CRTC1^270^ into the nucleus. (A)** Neurons expressing CRTC1^270^ were illuminated with a UV laser in the presence or absence (mock) of MNI-caged glutamate. The white dashed boxes indicate the region of activation (~10 um; ROA). Time-lapse images of the soma at every 20th frame (time stamp in parenthesis) are shown for CRTC1^270^ in both native green and photoconverted red channels. Scale bar = 20 μm. **(B–E)** Neurons expressing CRTC1^270^ were stimulated with **(B)** mock or MNI-glutamate in the presence or absence of APV and NBQX (^*^p = 0.49 mock vs. APV/NBQX; *p* < 0.0001 MNI vs. mock and APV/NBQX), **(C)** MNI-glutamate at 35°C or 23°C (^*^p < 0.0001 Mock 35°C vs. MNI 35°C, Mock 35°C vs. MNI 23°C, and MNI 35°C vs. MNI 23°C), **(D)** MNI-glutamate after incubation with NDZ (^*^p < 0.0001 DMSO vs. NDZ), or **(E)** pretreated with either BAPTA-AM or EGTA-AM prior to glutamate uncaging (^*^p < 0.0001 DMSO vs. EGTA vs. BAPTA and EGTA vs. BAPTA). Statistics were performed with linear regression analysis of the slopes for comparison of fit between data sets.

These findings encouraged us to undertake live-cell imaging of neurons expressing CRTC1^270^ (Figure 5). We previously showed that bath applied stimuli triggered nuclear accumulation of overexpressed CRTC1-Dendra2 (Ch'ng et al., 2012). Here we used CRTC1^270^ to label and visualize in real time the nuclear accumulation of CRTC1^270^ that is derived specifically from stimulated synapses. To do this, we transduced neurons with CRTC1^270^, incubated neurons in low Mg^2+^ Tyrode's solution in the presence of TTX and used UV illumination to simultaneously uncage glutamate and photoconvert CRTC1^270^ in a 10 μm dendritic region of activation (ROA) located approximately 100 μm away from the soma (Figure 5 and Supplementary Figure S4D). Calcium imaging using Fluo4AM revealed that local uncaging of glutamate triggered local elevations of intracellular calcium around the region of activation, which rapidly dissipated and did not reach the soma (Supplementary Figure S4E, Video S1). We first photoconverted CRTC1^270^ and uncaged glutamate in a single dendritic branch, and failed to detect nuclear accumulation of photoconverted CRTC1 (Supplementary Figure S4F). We then photoconverted CRTC1^270^ and uncaged glutamate in two ROAs located on adjacent dendritic branches and observed robust nuclear accumulation of photoconverted CRTC1 (Figure 5). We also optimized parameters for UV illumination to ensure maximal green to red photoconversion while minimizing photobleaching. This optimized protocol was used in all subsequent live imaging experiments.

Photoconverted (red) CRTC1^270^ was initially detected in the nucleus approximately 100–120 s (*t* = 10) after uncaging (Figure 5A), consistent with the speed of motor-driven active transport of vesicular structures in neuronal processes (0.8–1.2 μm/s; van den Berg and Hoogenraad, 2012). The nuclear accumulation of native (green) CRTC1^270^ signal is not surprising given that the UV stimulation at the ROA did not completely convert all the native green Dendra2 protein to red. Following that, we performed experiments to examine if local synaptic stimulation results in nuclear translocation of CRTC1^270^ and that the cell biological mechanisms underlying CRTC1^270^ were identical to those mediating the nuclear import of endogenous full-length CRTC1. As shown in Figure 5, we found that, like full-length CRTC1, BIC-induced nuclear import of CRTC1^270^ required both NMDA and AMPA receptors (Figure 5B), was temperature-sensitive (Figure 5C) and involved microtubule-dependent transport (Figure 5D). We also found that, like endogenous full-length CRTC1, CRTC1^270^ translocation from stimulated synapses to the nucleus was blocked by BAPTA but not EGTA, indicating that, like endogenous CRTC1, its nuclear translocation was triggered by local and not bulk elevations in Ca^2+^ at the plasma membrane (Figure 5E). Together, these findings indicate that CRTC1^270^ serves as a faithful reporter of stimulus-induced CRTC1 synapse to nuclear transport.

### CRTC1^270^ movement is biased toward the nucleus following neuronal stimulation

**Figure 6:**
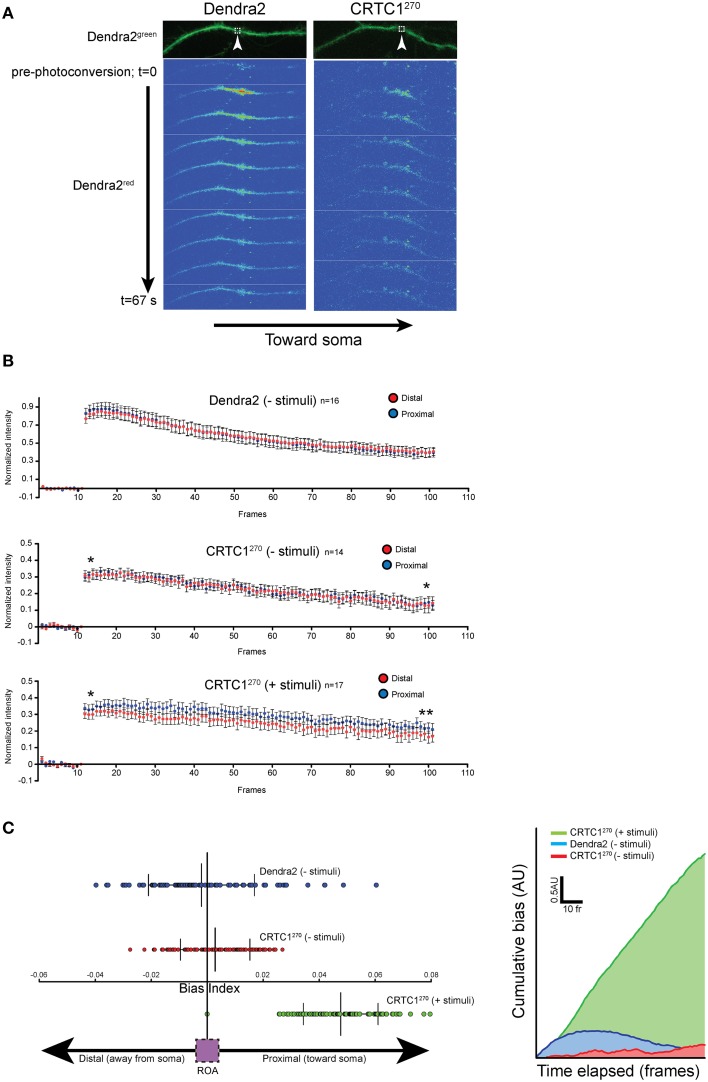
**Local CRTC1^270^ transport from dendrites is biased toward the nucleus during glutamatergic stimulation. (A,B)** Neurons expressing either Dendra2 alone or CRTC1^270^ were mock treated (-stimuli) or bath stimulated (+stimuli) followed by UV activation in a (1.5 um^2^) region (white box) of dendrite. The rapid movement of Dendra2^red^ was captured by spinning disc microscopy. The normalized intensity (over baseline levels) over time of Dendra2^red^ was quantified for a 40 μm segment of the dendrite either proximal (closer to the soma) or distal to the area of the photoconversion (upaired *t*-test, two-tailed ^**^p < 0.05 at *t* = 95–100; ^*^p > 0.05, not significant at *t* = 11–15). **(C)** The difference in pairwise spatial measurements of normalized Dendra2^red^ along the dendrite from the ROA was quantified as a bias index. A positive bias indicates a higher signal intensity in proximal dendrites. Data points were then plotted as a cumulative bias index over time elapsed for each stimulation paradigm (AU = arbitrary units).

As previous live-imaging studies of transport within dendrites have focused more on the movement of vesicles and organelles (Maeder et al., 2014), we set out to use the CRTC1^270^ reporter to visualize the retrograde transport of soluble molecules within dendrites. To achieve that, we visualized the transport of CRTC1 within a single dendritic branch, using spinning disc microscopy for rapid image acquisition (~100 ms/frame), which was required to detect the rapid translocation of CRTC1 within the dendrite. The signal we detected within the dendrite from local uncaging of glutamate was too weak to monitor by time lapse microscopy, and we thus combined local photoconversion of CRTC1^270^ with bath stimulation with BIC and forskolin (which promotes persistence of CRTC1 in the nucleus) to visualize the dynamics of a local population of photoconverted CRTC1, or, as a control, photoconverted Dendra2, within the dendrites of unstimulated and stimulated neurons. We tested multiple photoconversion parameters to optimize image acquisition and minimize photobleaching without compromising neuronal viability (Supplementary Figures S4G,H). In unstimulated conditions, photoconverted CRTC1^270^ underwent rapid bidirectional movement from the ROA, as did the control Dendra2 protein (Figure 6A). When neurons were stimulated, the movement of CRTC1^270^ became biased toward the soma (proximal), as detected by an increase in the average normalized intensity across the length of the dendrite proximal to the stimulation site over time (Figure 6B). We quantified the difference in normalized pixel intensity for pairwise length measurements (1.5 μm segments) along the distal and proximal lengths of the dendrite originating from the ROA and plotted a bias index. The majority of data points for stimulated CRTC1^270^ had a positive bias index, indicating a bias in movement toward the soma. Conversely, the non-stimulated CRTC1^270^ control had averages closer to zero, indicating a bidirectional distribution of the photoconverted signal across the length of the dendrite (Figures 6B,C). To better illustrate the shift in fluorescence intensity over time, we graphed the cumulative bias of CRTC1^270^ moving toward the soma during the length of the experiment (Figure 6C). A positive cumulative slope indicates that activated CRTC1^270^ concentrations are much higher on the proximal branch of the dendrite throughout image acquisition.

### Dephosphorylation at three conserved serine residues in CRTC1 triggers release of CRTC1 from 14-3-3ε and synapse to nucleus translocation

**Figure 7:**
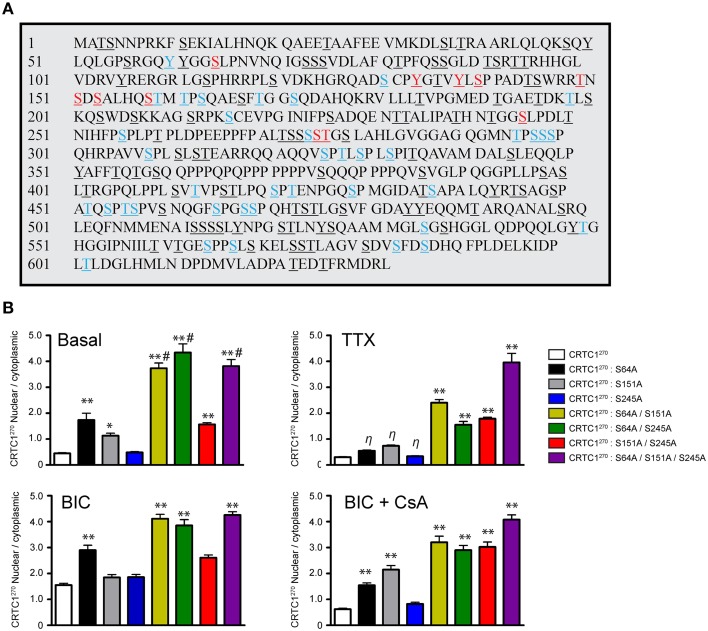
**Phosphorylation state of CRTC1. (A)** HA-tagged CRTC1 was immunoprecipitated from Neuro-2A cells and analyzed by mass spectrometry. Residues in both blue and red (50/643) were identified as phosphorylation sites. Phosphorylated sites labeled in red (13/50) share 100% conservation across 10 different species. All serine, threonine and tyrosines are underlined. **(B)** Single, double or triple CRTC1^270^ serine to alanine mutations were generated (S64A, S151A, and S245A) and expressed in neurons that were silenced with TTX, stimulated with BIC or BIC and CsA (^**^p < 0.001 relative to CRTC1^270^; ^*^p < 0.05 relative to CRTC1^270^; η: not significant relative to CRTC1^270^; ^#^*p* < 0.001 relative to S151A/S245A).

**Figure 8:**
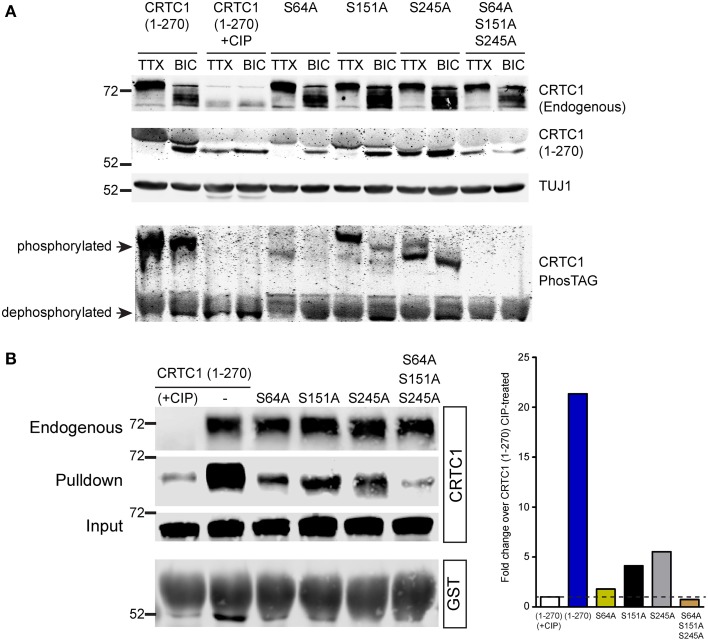
**Dephosphorylation of S64, S151 and S245 regulates interaction of CRTC1 with 14-3-3ε. (A)** The single and triple alanine CRTC1^270^ mutations described in Figure 7 were expressed in cortical neurons (21–28 DIV) and incubated with TTX or BIC. Neuronal lysates were analyzed via traditional or PhosTAG acrylamide gels and immunoblotted with antibodies against CRTC1 and TUJ1. **(B)** Cortical neuron expressing CRTC1^270^ point mutants were lysed and incubated with GST-purified 14-3-3ε and pulldowns were analyzed by immunoblotting with antibodies against GST or CRTC1. Endogenous CRTC1 binds to GST 14-3-3ε comparably across all samples while input controls indicate equivalent expression of all CRTC1^270^ proteins is neurons.

The phosphorylation state of CRTC1 undergoes complex changes after synaptic activity in rodent hippocampal neurons (Ch'ng et al., 2012; Nonaka et al., 2014). We hypothesized that the nuclear translocation of CRTC1 involved dephosphorylation of specific residues. Analysis of the mouse CRTC1 primary amino acid sequence revealed a high conserved sequence across multiple species with significant enrichment of serine and threonine residues (Figure 7A and Supplementary Figure S5A). To identify the residues that are phosphorylated in CRTC1, we transiently transfected mouse Neuro-2A cell lines with full-length HA epitope-tagged CRTC1 protein, purified the protein using HA antibodies and analyzed the samples by mass spectometry. We identified candidate phosphopeptides containing 50 putative phosphoresidues (33 Ser, 14 Thr and 3 Tyr residues; Figure 7A; labeled in color). This is likely an underestimate of the total number of phosphorylated residues in CRTC1 since we omitted phosphopeptide hits identified with less than 90% confidence levels. Sequence homology of CRTC1 indicated that 11 of the phosphorylated residues are 100% conserved across 10 species (Figure 7A; labeled in red). Nine of these are located within the N-terminal 270 amino acids. To begin to dissect the role of regulated dephosphorylation in the synapse to nucleus transport of CRTC1, we generated phosphoincompetent (serine to alanine) mutations at three of these sites, S64, S151, and S245, as single, double and triple mutants within CRTC1^270^. Dephosphorylation of S151 has been shown to be sufficient for nuclear import in non-neuronal cells (Kovács et al., 2007; Altarejos et al., 2008), while dephosphorylation of both S151 and S245 has been reported to be sufficient for significant CRTC1 nuclear accumulation in neurons (Nonaka et al., 2014). We also included S64 in our studies because, as shown in Figure 8, like S245, S64, shares 100% similarity across 13 species (with S64 being a T in *C. elegans*) and S64 and S245 have identical flanking amino acids (GGSLP), which are also highly conserved across species.

To elucidate the function of S64 and the conserved amino acids flanking S64, we made several substitution mutations at and surrounding S64 in CRTC1^270^, including a double tyrosine to alanine (Y60A/Y61A) mutant and a glutamine to alanine substitution mutant (Q70A; Supplementary Figure S5B) and expressed these mutant proteins in neurons. As shown in Figure 7B and Supplementary Figures S5B,D, the S64A mutant was cytoplasmically localized in TTX-silenced neurons, but exhibited enhanced nuclear accumulation compared to wildtype CRTC1^270^ in BIC-stimulated neurons and in control, unsilenced neurons (which have basal levels of action potential firing). Mutations at Y60, Y61, Q70 or at the conserved serine at 245 did not alter the localization of CRTC1^270^ as compared to wild type CRTC1^270^ in either silenced, basal or stimulated neurons.

We previously reported that unlike in non-neuronal cells (Altarejos et al., 2008), CRTC1 bearing a S151A mutation did not result in constitutive CRTC1 nuclear accumulation. We generated the S151A mutation for CRTC1^270^, and again found that when expressed in neurons, the mutant localized to the cytoplasm in TTX-silenced neurons. Although we observed significantly more S151A-CRTC1^270^ in the nucleus in unsilenced neuronal cultures, we did not detect any enhancement of nuclear accumulation in BIC-stimulated neurons (Figure 7). Similarly, a single serine to alanine mutation at S245 also did not result in constitutive accumulation of CRTC1^270^ in the nucleus. These results indicate that dephosphorylation of S151 alone, similar to S64 and S245 is not sufficient to promote robust nuclear import of CRTC1.

Since none of the single serine to alanine mutations generated a constitutive nuclear localization of CRTC1^270^ in TTX-silenced neurons, we tested the possibility that dephosphorylation at combinations of the three sites was required for nuclear accumulation. To test this idea, we generated double and triple serine to alanine substitution mutants (S64A/S151A; S64A/S245A; S151A/S245A; and S64A/S151A/S245A) in CRTC1^270^. We found that all three double mutants exhibited increased nuclear accumulation of CRTC1^270^ in TTX-silenced neurons, and enhanced nuclear accumulation in basal and BIC-stimulated neurons. The amount of CRTC1^270^ nuclear accumulation in the double mutants was less than in the triple S64A/S151A/S245A mutant under all conditions. Thus, when all three serine residues were simultaneously mutated to alanine, CRTC1^270^ was constitutively localized in the nucleus (Figure 7 and Supplementary Figure S5B). We also constructed serine to aspartic acid or glutamic acid phosphomimetic mutations at S64, S151, and S245 individually but found that none of these mutations constitutively localized CRTC1 to the cytoplasm (Supplementary Figures S5B–D).

We used a combination of conventional and PhosTag immunoblotting to monitor the effect of these mutations on the mobility of CRTC1^270^. The mobility of CRTC1^270^ was higher in TTX silenced than in BIC-stimulated neurons, with phosphatase treatment of the lysates dropping the molecular weight (MW) of CRTC1^270^ similar to the MW observed in BIC-stimulated neurons (Figure 8A). Of the three single mutations, only the S245A mutation showed significant amounts of the lowest MW band in TTX stimulated neurons, though PhosTag gel analysis indicated that the S245 protein was not completely dephosphorylated (Figure 8A). One possible explanation is that S245A mutation resulted in subsequent dephosphorylation at multiple sites on CRTC1, resulting in a lower molecular weight band. All three serine to alanine mutants were responsive to BIC stimulation, as BIC increased the concentration of the lowest MW species. In contrast, the triple mutant S64A/S151A/S245A looked like the phosphatase-treated sample, with only the lowest MW band in both TTX-silenced and BIC-stimulated neurons (Figure 8A). As our earlier 2-D gel analyses revealed a large number of CRTC1 species, each with differential patterns of phosphorylation (Ch'ng et al., 2012), the results shown in Figure 8A suggest that dephosphorylation of S64, S151, and S245 may function to trigger subsequent differential dephosphorylation throughout CRTC1.

We previously demonstrated that CRTC1 binds to 14-3-3ε in an activity-dependent manner (Ch'ng et al., 2012). When neurons were silenced with TTX, phosphorylated CRTC1 bound 14-3-3ε and was sequestered in the cytoplasm; BIC triggered CRTC1 dephosphorylation and dissociation from 14-3-3ε. To assess whether S64, S151 or S245 play roles in anchoring CRTC1 to 14-3-3ε, we transduced cultured cortical neurons with lentiviral vectors expressing CRTC1^270^ harboring substitution mutations (S64A, S151A, S245A, or S64A/S151A/S245A), collected neuronal lysates and incubated with 14-3-3ε-GST. As shown in Figure 8B, single point mutations at S64, S151 and S245 diminished but did not abolish the interaction between CRTC1 and 14-3-3ε. Notably, the degree of interaction between the mutants and 14-3-3ε correlated inversely with their nuclear accumulation (i.e., S64A had the highest nuclear accumulation but lowest interaction with 14-3-3ε Figure 8). The triple S64A/S151A/S245A mutant did not bind 14-3-3ε similar to phosphatase-treated CRTC1^270^ (Figure 8B).

## Discussion

CRTC1 undergoes robust nuclear translocation during learning-related synaptic plasticity (Zhou et al., 2006; Kovács et al., 2007; Ch'ng et al., 2012; Nonaka et al., 2014) but the mechanism for this activity-dependent translocation remains elusive. Our goal was to further characterize the cell biology of activity-dependent CRTC1 synapse to nucleus transport. Our studies revealed that local activation of synaptic glutamatergic receptors and L-type VGCCs generate the source of calcium required for the regulated nuclear import of CRTC1. We also showed that CRTC1 undergoes dynein-mediated and NLS-dependent transport along microtubules to reach the nucleus. Next, we identified the regions of CRTC1 that are critical for regulated trafficking of CRTC1, including three serine residues that undergo dephosphorylation to be released from 14-3-3ε at the synapse. Finally, we developed a reporter containing the minimal regions of CRTC1 required for regulated nuclear import fused to the photoconvertible fluorescent protein Dendra2, and used this to monitor in real time, stimulus-induced synapse to nucleus transport of CRTC1 in neurons.

CRTC1 nuclear translocation has been shown to depend on elevations in intracellular calcium resulting from synaptic activation (Zhou et al., 2006; Kovács et al., 2007; Ch'ng et al., 2012; Nonaka et al., 2014). In this report, we dissected the contributions of specific channels and receptors in generating the elevations in calcium required for CRTC1 nuclear translocation. We found that inhibition of AMPA-type receptors blocks CRTC1 synapse to nucleus translocation, but that calcium entry through AMPA receptors along with activation of L-type VGCC was sufficient to promote CRTC1 nuclear import. In other experiments, we found that calcium influx through NMDA receptors is sufficient to trigger CRTC1 nuclear translocation as long as there is sufficient depolarization to relieve the Mg^2+^ block in the receptor (Figure 1). Recent studies indicate that depolarization activates L-type VGCC to couple local calcium influx with transcription in the nucleus (Wheeler et al., 2012) in a manner that involves calcineurin-mediated dephosphorylation of CamKIIγ, which in turn transports calcium-calmodulin to the nucleus where it activates CamKIV, leading to the phosphorylation of CREB (Ma et al., 2014). Here we find that either activation of NMDA receptors in the absence of L-type VGCC activation, or AMPA receptor activation coupled with L-type VGCC activation is sufficient to promote CRTC1 synapse to nucleus translocation, suggesting that CRTC1-mediated transcription, like that of NFkB (Meffert et al., 2003) and Jacob (Karpova et al., 2013), tracks synaptic stimulation rather than cell-wide neuronal depolarization.

Comparison of figures magnitude of the activity-dependent nuclear accumulation of CRTC1 varied somewhat between experiments (see Figures 2, 5). This can be attributed to difference in the basal network activity of cultures, since cultures with high levels of basal activity have increased concentrations of CRTC1 in the nucleus. Of relevance, we note that each set of experiments performed in this study include negative (TTX) and positive (bicuculline) controls for CRTC1 nuclear accumulation.

A paucity of cell biological studies address the long-distance retrograde transport of soluble proteins in dendrites. Theoretical evidence suggest that fast local signaling can be mediated by diffusion only under short distances (< 200 nm); (Kholodenko, 2003; Howe, 2005). In this paper, we show that CRTC1 is actively transported to the nucleus in an energy-dependent manner that requires microtubules and is mediated by the motor protein dynein (Figure 2). While dynein is a major microtubule-based molecular motor for cargo transport from soma to dendrites (Kapitein et al., 2010), its role in synapse to nucleus signaling is not well characterized. Since microtubules have mixed polarity in dendrites (Silverman et al., 2010), our studies support a critical role for the minus-end directed motor dynein in mediating the retrograde transport of CRTC1 from synapse to nucleus.

Unlike movement of large organelles or vesicular trafficking, studying the transport of soluble proteins such as CRTC1 in dendrites poses a greater challenge since the protein moves diffusely throughout the dendrite rather than as a punctate vesicular structure that can be tracked. By fluorescently labeling a subpopulation of CRTC1 in dendrites via photoconversion of Dendra2, we were able to track and quantify the movement of the soluble protein as a fluorescent “plume” as it propagated along dendrites and to monitor the accumulation of photoconverted Dendra2 signal in the nucleus. We detected a small but significant bias of retrograde movement of CRTC1 toward the soma following stimulation. It is likely that the small ROA coupled with the relative abundance of fluorescent CRTC1 in dendrites decreases signal to noise and thus reduced our ability to detect a larger magnitude bias in retrograde movement toward the soma. At present, it is unclear if soluble proteins in dendrites are transported as a single protein or as a macromolecular signaling complex associated with motor proteins. In axons, the hypothesis that bulk movement of soluble proteins is mainly transported as “slow” axoplasmic flow has been recently challenged by observations that soluble proteins can assemble into higher order structures that engage the active transport machinery for axonal transport (Scott et al., 2011).

We chose to study CRTC1^270^ instead of the full length protein because our goal was to identify the minimal region and the signals contained within this region that drives activity-dependent nuclear translocation and for the practical reason that tagged full-length CRTC1 was too large to package in neurotrophic viral vectors. By excluding the carboxy-terminal transcriptional activation domain of the protein, we also avoided any aberrant transcription that might occur due to overexpression of CRTC1^270^. We systematically tested CRTC1^270^ and found it responds to stimuli and translocates to the nucleus similar to wild type protein, that it does not act as a dominant negative inhibitor of CREB-mediated transcription, and that lentiviral-mediated expression under control of the *Camk2*α promoter does not affect the long term viability of mature neurons. Our results suggest that CRTC1^270^ is a faithful reporter of neuronal activity and can be used as a tool for studying synapse to nuclear signaling in *in vivo* preparations, where its nuclear translocation may be used to rapidly identify neurons that are activated in response to specific stimuli. Moreover, our results suggest that CRTC1^270^ has the potential to serve as a useful tool for activity-dependent delivery of molecules to the nucleus. Thus, one could couple CRTC1^270^ to a chimeric transcriptional regulator, and deliver it to the nucleus only following glutamatergic activity.

CRTC1 is one of several post-synaptically localized proteins that undergo nucleocytoplasmic shuttling during various forms of neuronal plasticity (Lee et al., 2007; Lai et al., 2008; Marcora and Kennedy, 2010; Sekeres et al., 2012; Karpova et al., 2013). Many of these proteins contain strong NLSs that engages the heterodimeric importin α/β1 classical nuclear adaptor protein complex that couples to dynein-mediated microtubule transport to enter the nucleus (Perlson et al., 2005; Mikenberg et al., 2007; Shrum et al., 2009; Ben-Yaakov et al., 2012). We have identified a highly conserved and potent NLS in CRTC1 that is both necessary and sufficient to trigger nuclear entry. However, while CRTC1 is actively transported to the nucleus, its entry is not mediated by the classical nuclear import pathway. CRTC1 may enter the nucleus by binding to other NLS-bearing proteins or by non-conventional nuclear import pathways such as via a member of the importin β superfamily (Chook and Süel, 2011), direct binding to nuclear pore complex (Koike et al., 2004) or it may be escorted by other nuclear chaperone proteins (Fagotto et al., 1998).

We discovered three residues, S64, S151, and S245 that play crucial and synergistic roles in nuclear accumulation of CRTC1. While individual point mutations at these serine residues did not significantly alter the localization of CRTC1 in silenced neurons, a triple phospho-incompetent mutation resulted in constitutive nuclear localization in the absence of neuronal activity (Figure 8). Since the nuclear accumulation of the phosphorylation-incompetent mutants inversely correlated with the association with 14-3-3ε cytoplasmic anchoring protein, it is likely that the constitutive transport of CRTC1 into the nucleus, as opposed to defects in nuclear export, underlies the robust nuclear accumulation of the phospho-incompetent mutants.

Nonaka and colleagues recently reported that alanine scanning mutations in S151 and S245 resulted in constitutive nuclear localization of CRTC1 in cortical neurons (Nonaka et al., 2014). However, we found that a S151 and S245 double mutation showed only a slight enhancement of nuclear accumulation over the wild type protein in silenced neurons. In contrast, we provide evidence that phosphorylation at S64 is a potent regulator of CRTC1 nuclear translocation. In unstimulated neurons, a single point mutation at S64 alone led to a 2-fold increase in the nuclear to cytoplasmic ratio of CRTC1 while a double mutation of S64 paired with either S151 or S245 resulted in a 4-5-fold increase in the nuclear accumulation of CRTC1. Taken together, our results indicate that the phosphorylation state of S64 is a major contributor, together with S151 and S245, in regulating CRTC1 nucleocytoplasmic shuttling in neurons.

The fact that dephosphorylation of only 3 amino acids is sufficient to trigger nuclear import of CRTC1 raises questions about the function of the large number of remaining, highly conserved phosphorylated residues. It is unclear if dephosphorylation of any of these 3 amino acids, either individually or as a combination, also results in a coordinated dephosphorylation at other phosphorylation sites. Given that CRTC1 exists in multiple phosphorylated forms as assessed by two dimensional gel analysis (Ch'ng et al., 2012), it is plausible that the activation of distinct signaling pathways within dendrites and spines following specific types of stimulation can differentially alter the pattern of CRTC1 phosphorylation which may serve as a code that couples patterns of stimulation with specific programs of gene expression.

Taken together, the results of our experiments provide insight into the mechanisms by which soluble signals are transported from the synapse to the nucleus during transcription-dependent activity. They provide molecular insight not only into how activity regulates CRTC1 to promote its nuclear import, but also into some of the cell biological pathways mediating this long-distance transport.

## Funding

The work was supported by a NARSAD Young Investigator Award (to TC) and NIH R01 MH MH077022 (to KM).

### Conflict of interest statement

The authors declare that the research was conducted in the absence of any commercial or financial relationships that could be construed as a potential conflict of interest.
