# Supplementary material for: Cell biological mechanisms of activity-dependent synapse to nucleus translocation of CRTC1 in neurons
Source: Front Mol Neurosci. 2015 Sep 4;8:48. doi: 10.3389/fnmol.2015.00048 (PMC4560099; doi:10.3389/fnmol.2015.00048)
Supplement: Supplementary file 2 [file DataSheet1.PDF]

# *Supplementary Material*

# Cell Biological Mechanisms of Activity-Dependent Synapse to Nucleus Translocation of CRTC1 in Neurons.

**Toh Hean Ch'ng*, Martina DeSalvo, Peter Lin, Ajay Vashisht, James A. Wohlschlegel, and Kelsey C. Martin***

*** Correspondence: Toh Hean Ch'ng**: thchng@ntu.edu.sg, kcmartin@mednet.ucla.edu

## 1. Supplementary Data

### 1.1 Video: Calcium transients in hippocampal neurons during glutamate uncaging.

Time-lapse imaging of cultured hippocampal neurons loaded with Fluo4AM calcium dye and subjected to repeated glutmate uncaging. Each burst of green signal represents a spike in intracellular calcium concentrations as described in methods and materials and quantified in Appendix fig S4. Images were collected at 1.59 frames / s for 80 frames and compressed and replayed at 10 frames/s.

## 2. Supplementary Figures and Tables

### 2.1. Supplementary Figures

### Supplementary Figure S1

(A)

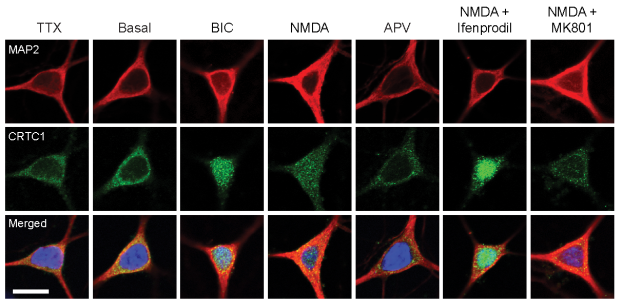


(B)

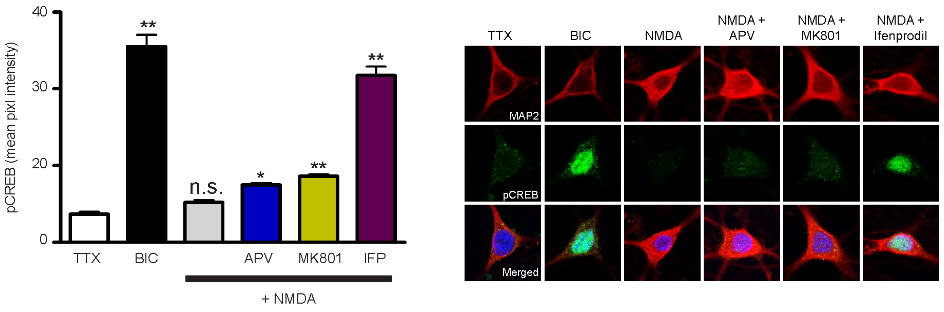

(C)

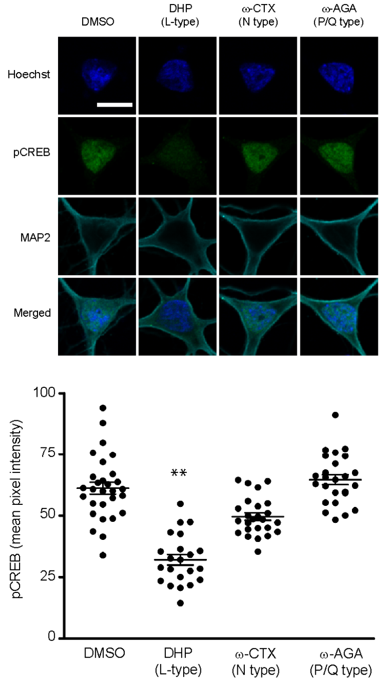


(D)

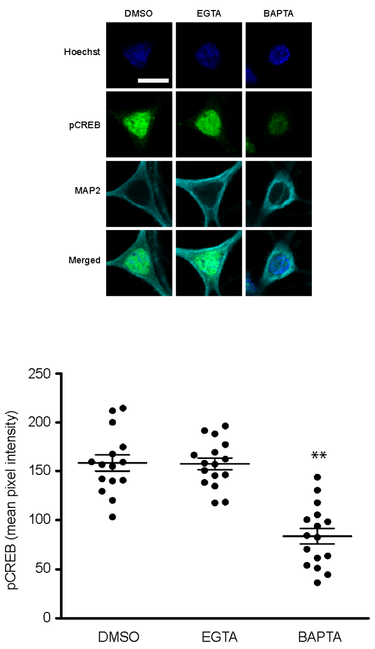


**Supplementary Figure S1. CREB phosphorylation (S133) requires activation of NMDA receptors and L-type VGCC.**

(A) Neurons were treated as described in figure 1A and immunostained with antibodies against CRTC1, MAP2 and Hoechst nuclear dye (blue). (B) Neurons were incubated with TTX, IFP or MK801 before being stimulated with NMDA in the presence of the receptor antagonists. Neurons were immunolabeled with antibodies against pCREBS133, MAP2, and Hoechst nuclear dye (blue). The mean pixel intensity of pCREB in the nucleus was quantified and graphed (**p<0.001, *p<0.05 relative to TTX-silenced). (C) Mean nuclear intensity of pCREB133 in neurons were quantified after depolarization with KCl in the presence of NIM, conotoxin (ω-CTX), agatoxin (ω-AGA) or mock treated with DMSO (** p<0.001 relative to DMSO). (D) Membrane permeable calcium chelators BAPTA and EGTA were incubated in neurons prior to depolarization with KCl. Mean nuclear intensity of pCREB133 in neurons were quantified (** p<0.001 relative to DMSO). All scale bars = 10 µm.

**Supplementary Figure S2**

(A)

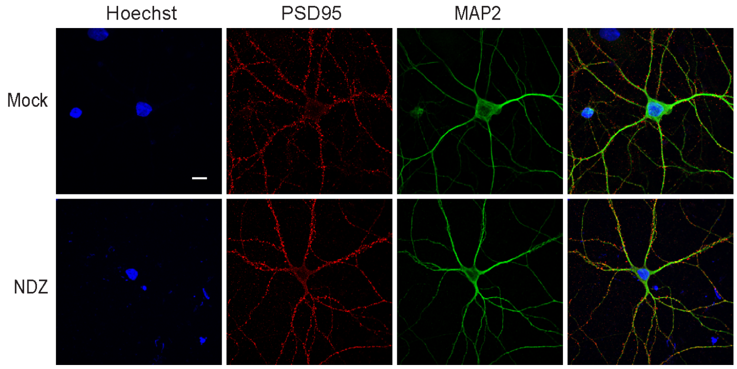

(B)

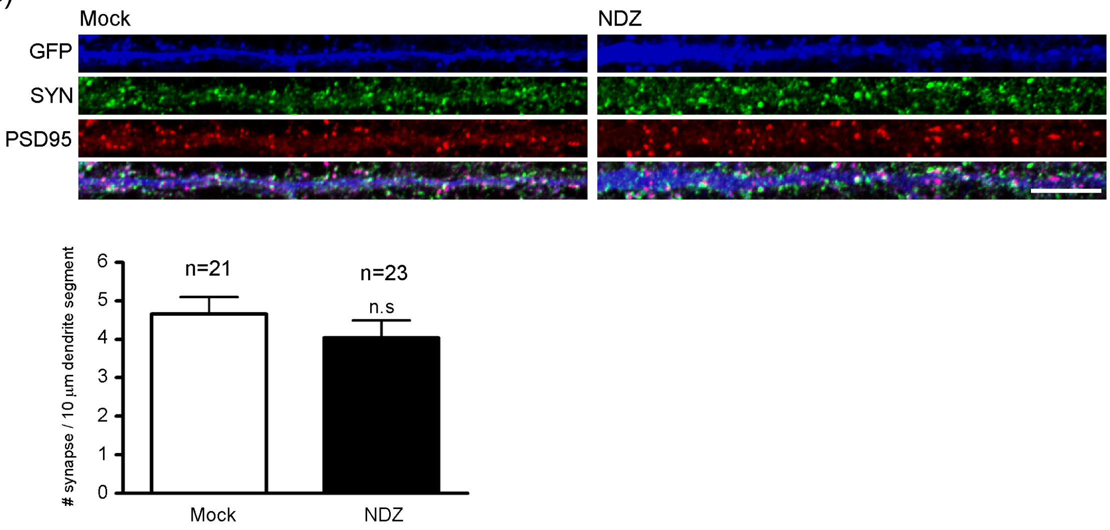


(C)

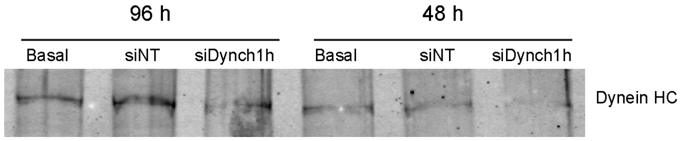


**Supplementary Figure S2. CRTC1 is actively transported to the nucleus.**

(A) Neurons were incubated with either DMSO or NDZ before being immunolabeled with antibodies against MAP2 (green), PSD95 (red) and Hoechst nuclear dye (blue). (B) Neurons were transduced with AAV expressing GFP (blue) driven under the neuron-specific synapsin promoter and treated with DMSO (mock) or NDZ. Neurons were then immunostained with synapsin (SYN) and PSD95 antibodies. The number of synapses per 10 µm segment of dendrites was quantified (refer to methods and materials). (C) Mouse hippocampal neurons were incubated with siRNA targeted against dynein heavy chain (siDynch1h) or non-targeted control (siNT). Neurons lysates were immunoblotted with antibodies against the dynein heavy chain.

**Supplementary Figure S3**

(A)

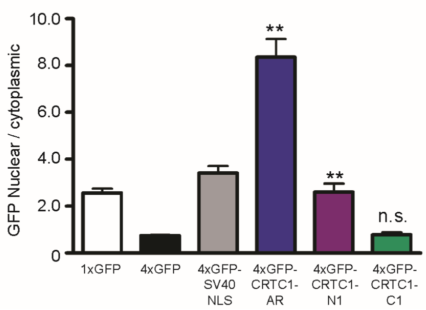


(B)

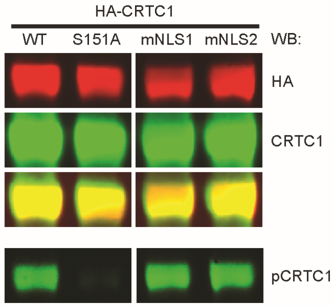

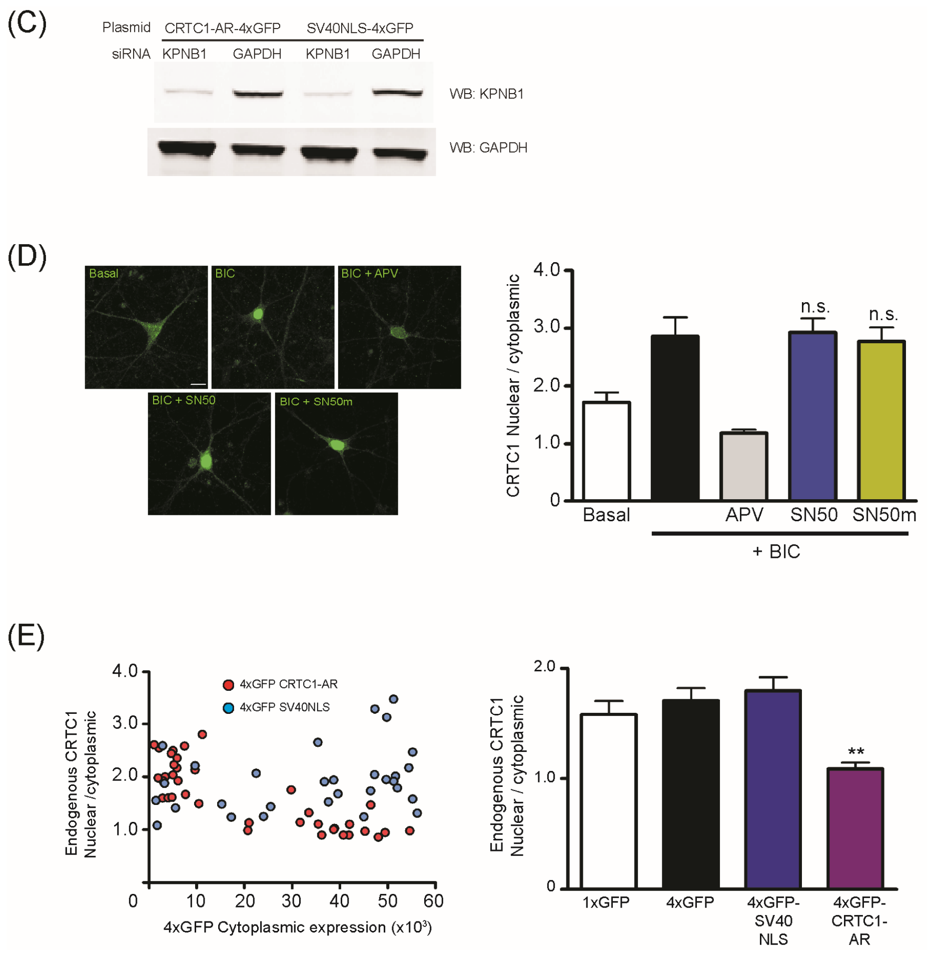


**Supplementary figure S3. CRTC1 encodes an arginine-rich NLS that does not engage the classical nuclear import pathway.**

(A) HEK293T cells were transfected with 4xGFP fusion constructs as described in Figure 3. The GFP signal in the nucleus and cytoplasm was quantified (** $p<0.001$ relative to 4xGFP; * not significant relative to SV40NLS but $p<0.001$ relative to 4xGFP; n.s. not significant relative to 4xGFP). (B) HEK293T cells were individually transfected with plasmids expressing i) HA-tagged wildtype CRTC1 (WT), ii) HA-tagged S151A CRTC1 mutant (S151A) or iii) HA-tagged CRTC1 nuclear localization mutants (mNLS1) and (mNLS2). Immunoblotting with antibodies specific for the HA-epitope, CRTC1 or phosphorylated CRTC1 at residue 151 (pCRTC1) was performed. (C) HEK293T cells were transfected with CRTC1-AR-4xGFP or SV40NLS-4xGFP after incubation with siRNA against KPNB1 or GAPDH. Lysates were immunoblotted for KPNB1 or GAPDH and quantified. KPNB1 siRNA decreased importin β1 protein concentration by 45% compared to cells incubated with GAPDH siRNA. (D) Neurons were incubated with membrane-permeable NLS peptide (SN50) or a mutant NLS peptide (SN50m) and stimulated with BIC (n.s. not significant relative to BIC only). (E) Neurons expressing 4xGFP-CRTC1-AR or 4xGFP-SV40NLS were stimulated with BIC and the endogenous CRTC1 with the corresponding total intensity of 4xGFP in the nucleus and cytoplasm of each neuron were quantified. Neurons where 4xGFP signal is overexpressed (>20, 000 total fluorescence units) were selected and quantified for endogenous CRTC1 (** $p<0.001$ relative to 4xGFP-SV40NLS).

## Supplementary Figure S4

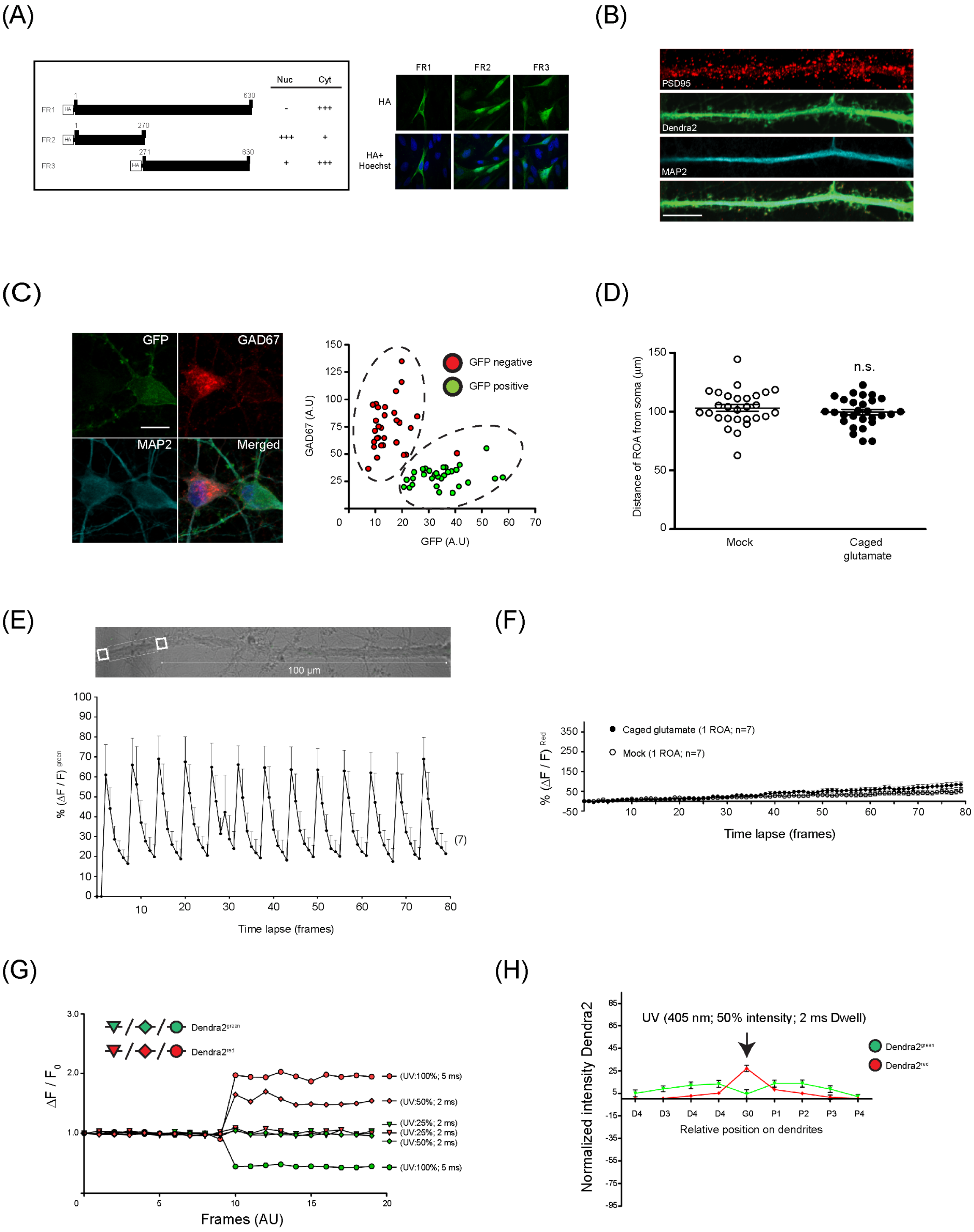


**Supplementary Figure S4. Time-lapse imaging of CRTC1$^{270}$ translocation in neurons.**

(A) The nuclear and cytoplasmic localization of HA-tagged full length CRTC1 (FR1), CRTC1 amino terminal fragment (FR1: 1-270) and carboxy-terminal fragment (FR3: 271-630) were qualitatively assessed in Chinese Hamster Ovary (CHO) cell culture. (B) Neurons expressing CRTC1$^{270}$ were

incubated with TTX and immunostained with antibodies for PSD95, MAP2 and Dendra2. A magnified image of a dendrite is shown. (C) Neurons expressing farnesylated GFP driven under the expression of a *Camk2α* promoter were fixed and immunostained. The mean pixel intensity of GFP and GAD67 in randomly selected neuronal cell bodies was quantified. Two populations of neurons were identified and highlighted by dashed ellipses. One group with robust GFP expression has low levels of GAD67 signal. Conversely, neurons with robust GAD67 signal have little to no GFP expression. (D) Neurons were transduced and treated as described in figure 5 for time-lapse imaging. Two regions of activation where the dendrites are exposed to UV illumination (ROA; white dashed box in figure 5) were selected approximately 100 μm away from the soma. The distance of the ROA from the soma was measured for each experimental condition and plotted on a bar graph (n.s. not significant). (E) Neurons loaded with Fluo4AM and calcium transients were recorded upon glutamate uncaging (Video S1). The white box (dashed) indicates the ROA in dendrites (approx. 100 μm away from the cell body) subjected to UV excitation to uncage glutamate. To quantify the change of Fluo4AM fluorescence in dendrites, the average intensity of two regions (white box; solid) flanking the ROA was quantified and plotted as a line graph showing the change of fluorescence compared to baseline values over time. (F) Neurons expressing $CRTC1^{270}$ were imaged as described in figure 5 except that UV activation is targeted to only a single dendritic branch (1 ROA). The amount of nuclear $Dendra2^{red}$ signal was quantified and plotted. (G) To ensure optimal photoconversion without photobleaching, neurons were transduced with a lentivirus expressing Dendra2 for 1 wk before being fixed and placed in the same chamber setup used for time-lapse imaging of neurons. Using the same live-cell imaging methodologies, the neuronal soma was subjected to varying intensities of UV (405 nm) excitation as well as variable laser dwell times (2 ms-5 ms). The intensity of the native Dendra2 green emission as well as the photoconverted red emissions were imaged and quantified. (H) To ensure that local photoconversion of Dendra2 in dendrites is localized precisely within the boundaries of the ROA (2.25 $\mu m^2$), fixed neurons were UV stimulated as described above (50% UV intensity; 2 ms dwell time). The normalized intensities of both the native green and photoconverted red Dendra2 intensities were calculated every 10 μm (up to 40 μm) from the point of photoconversion (G0) in the dendrites to either proximal (P1 to P4) or distal (D1-D4) to the site of photoconversion. Data reported as mean ± SEM.

## Supplementary Figure S5

(A)

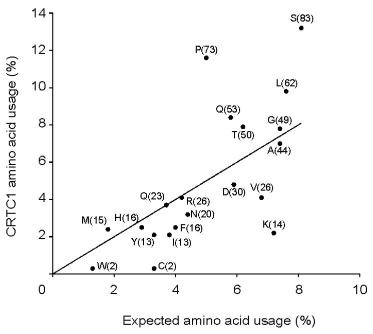

1 MATSNNPRKF SEKIALHNQK QAEETAAFEE VMKDLSLTRA ARLQLQKSQY
51 LQLGPSRGQY YGGSLPNVNQ IGSSSVDLAF QTPFQSSGLD TSRTTRHHGL
101 VDRVYRERGR LGSPHRRPLS VDKHGRQADS CPYGTVYLSP PADTSWRRTN
151 SDSALHQSTM TPSQAESFTG GSQDAHQKRV LLLTVPGMED TGAETDKTLS
201 KQSWDSKKAG SRPKSCEVPG INIFPSADQE NTTALIPATH NTGGSLPDLT
251 NIHFPSPLPT PLDPEEPPFP ALTSSSSTGS LAHLGVGGAG QGMNTPSSSP
301 QHRPAVVSPL SLSTEARRQQ AQQVSPTLSP LSPITQAVAM DALSLEQQLP
351 YAFFTQTGSQ QPPPQPQPPP PPPPVSQQQP PPPQVSVGLP QGGPLLPSAS
401 LTRGPQLPPL SVTVPSTLPQ SPTENPGQSP MGIDATSAPA LQYRTSAGSP
451 ATQSPTSPVS NQGFSPGSSP QHTSTLGSVF GDAYYEQQMT ARQANALSRQ
501 LEQFNMMENA ISSSSLYNPG STLNYSQAAM MGLSGSHGGL QDPQQLGYTG
551 HGGIPNIILT VTGESPPSLS KELSSTLAGV SDVSFDSDHQ FPLDELKIDP
601 LTLDGLHMLN DPDMVLADPA TEDTFRMDRL

Sequence conservation among 10 species: 100%, 90%, 80%, 70%, 60%, <50%

(B)

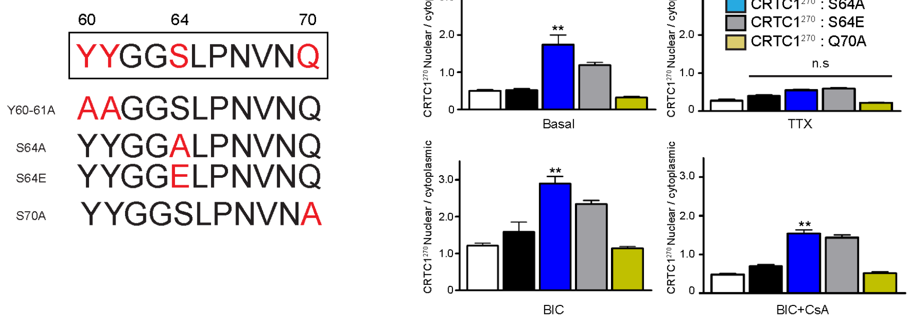


(C)

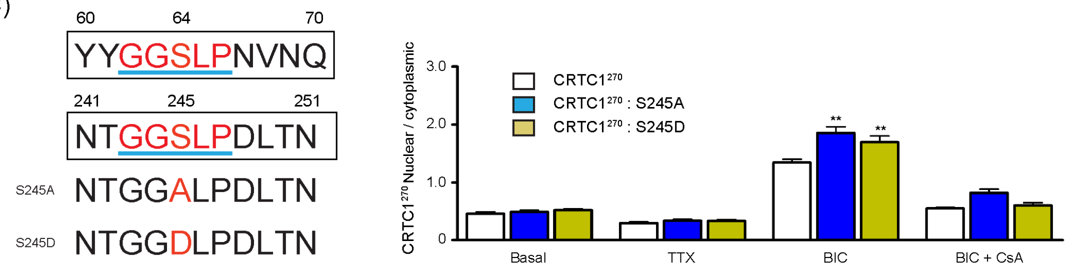


(D)

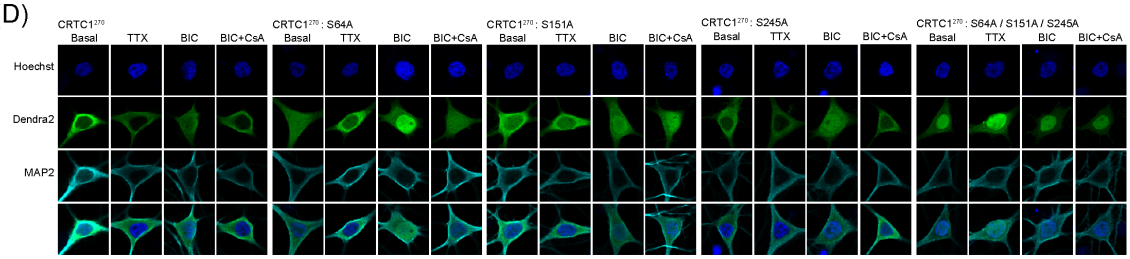

**Supplementary Figure S5. Phosphorylation state of CRTC1.**

(A) Scatter plot showing expected and observed CRTC1 amino acid usage. Brackets after each single letter amino acid code indicates the number of that particular residue present in CRTC1. Any amino acid that falls above the perfect correlation line indicates over representation of that amino acid in CRTC1. Conservation of phosphorylated residues between 10 different species (human, mouse, rat, zebrafish, puffer fish, opossum, xenopus, chicken, dog, horse). The color-coded scale indicates the degree of conversation with amino acids labeled in red showing 100% conservation across 10 species compared. (B) $CRTC1^{270}$ serine to alanine mutations were created in the region flanking the conserved amino acid S64 which includes Y60A/Y61A, S64A, S64E and Q70A. These constructs were expressed in neurons stimulated with TTX, BIC or BIC in the presence of CsA (** $p<0.001$ relative to $CRTC1^{270}$ for each treatment condition). (C) Neurons were transduced with lentivirus expressing a serine to aspartic acid phosphomimetic mutation at S245. The neurons were then stimulated and processed for immunocytochemistry as described in (A) (** $p<0.001$ relative to $CRTC1^{270}$). (D) Confocal micrographs of neurons expressing either $CRTC1^{270}$ or the serine to alanine mutants (single and triple) and stimulated as described in figure 7.
